# Mechanisms of gap gene expression canalization in the Drosophila blastoderm

**DOI:** 10.1186/1752-0509-5-118

**Published:** 2011-07-28

**Authors:** Vitaly V Gursky, Lena Panok, Ekaterina M Myasnikova, Manu, Maria G Samsonova, John Reinitz, Alexander M Samsonov

**Affiliations:** 1 Theoretical Department, Ioffe Physical-Technical Institute of the Russian Academy of Sciences, St. Petersburg, 194021 Russia; 2 Department of Applied Mathematics and Statistics, and Center for Developmental Genetics, Stony Brook University, Stony Brook, NY 11794-3600, USA; 3 Chicago Center for Systems Biology, and Department of Ecology and Evolution, The University of Chicago, Chicago, IL 60637, USA; 4 Department of Computational Biology, Center for Advanced Studies, St. Petersburg State Polytechnical University, St. Petersburg, 195259 Russia; 5 Department of Statistics, and Department of Molecular Genetics and Cell Biology, The University of Chicago, Chicago, IL 60637, USA

## Abstract

**Background:**

Extensive variation in early gap gene expression in the *Drosophila *blastoderm is reduced over time because of gap gene cross regulation. This phenomenon is a manifestation of canalization, the ability of an organism to produce a consistent phenotype despite variations in genotype or environment. The canalization of gap gene expression can be understood as arising from the actions of attractors in the gap gene dynamical system.

**Results:**

In order to better understand the processes of developmental robustness and canalization in the early *Drosophila *embryo, we investigated the dynamical effects of varying spatial profiles of Bicoid protein concentration on the formation of the expression border of the gap gene *hunchback*. At several positions on the anterior-posterior axis of the embryo, we analyzed attractors and their basins of attraction in a dynamical model describing expression of four gap genes with the Bicoid concentration profile accounted as a given input in the model equations. This model was tested against a family of Bicoid gradients obtained from individual embryos. These gradients were normalized by two independent methods, which are based on distinct biological hypotheses and provide different magnitudes for Bicoid spatial variability. We showed how the border formation is dictated by the biological initial conditions (the concentration gradient of maternal Hunchback protein) being attracted to specific attracting sets in a local vicinity of the border. Different types of these attracting sets (point attractors or one dimensional attracting manifolds) define several possible mechanisms of border formation. The *hunchback *border formation is associated with intersection of the spatial gradient of the maternal Hunchback protein and a boundary between the attraction basins of two different point attractors. We demonstrated how the positional variability for *hunchback *is related to the corresponding variability of the basin boundaries. The observed reduction in variability of the *hunchback *gene expression can be accounted for by specific geometrical properties of the basin boundaries.

**Conclusion:**

We clarified the mechanisms of gap gene expression canalization in early *Drosophila *embryos. These mechanisms were specified in the case of *hunchback *in well defined terms of the dynamical system theory.

## Background

Development is surprisingly robust to environmental stress, intrinsic fluctuations, and genetic variability in populations. These facts, together with the observation that cell type is a discrete rather than continuous property, led C. H. Waddington to propose that developmental processes have innate error-correction properties, which he called "canalization" [1]. Waddington visualized error correction in terms of an "epigenetic landscape," in which the developmental state of an organism is analogous to a ball rolling down a sloping landscape containing multiple "hills" and "valleys": as development progresses, cells take different paths down this landscape and so adopt different fates. Uncontrolled differentiation does not occur because the hills act as barriers and the state remains near a valley floor. This picture has natural corollaries in terms of genetic variability and evolution. Although the shape of the landscape may alter slightly in the face of genetic variation, the tendency of the system to stay near the valley floor will buffer the phenotypic consequences of these variations. Even under larger changes over evolutionary time, the tendency of the system to stay close to the valley floors will preserve and buffer developmental pathways in the face of evolutionary change.

We recently demonstrated the existence of canalization at the molecular level in the segment determination system of the fruit fly *Drosophila melanogaster *[2]. We showed that with respect to the gap gene system, canalization was a consequence of gap gene cross-regulation [3], and furthermore that it was associated with error correction by dynamical attractors [4], a precise mathematical formulation of Waddington's ideas about hills and valleys [5]. That work remains incomplete for reasons involving both broad biological considerations and specific mathematical points. With respect to large scale biological issues, the fact that canalization is under the control of natural selection means that all regulatory systems in an organism are in some sense selected for canalizing properties. In a general evolutionary context, canalization tends to appear as a multigenic trait involving the buffering of underlying genetic variation [6-8]. In a specific example concerning the segmentation system, it was shown that the differing proportional placement of *even-skipped *stripes in three lines and two species of *Drosophila *[9] depended on differences in the maternally expressed genes of flies in these lines, rather than the zygotic gap genes, the cross-regulation of which we have shown to ensure proportional spacing [3] because of the alternating arrangement of strongly mutually repressing gap gene expression domains [10].

In comparing our results to those of Lott and coworkers, we note that we considered the canalizing behavior of a specific zygotic component of the segmentation system, while the study of Lott et al. looked at the canalizing behavior of the full set of organismal genes, many subsets of which are presumably engaging in their own form of canalizing behavior. Closing the gap between the genetic control of canalization at the level of populations versus the well defined small networks of genes considered in developmental genetics will require finding the specific genes responsible for population effects as well as taking the complementary step of incorporating additional mechanisms and genes into the well characterized systems arising in a developmental genetics context.

In [3,4], we used the "gene circuit" approach to demonstrate that the reduction in variance of gap gene expression was a consequence of gap gene cross regulation. Gene circuits [11-13] are dynamical models that can reproduce observed gene expression patterns by reconstituting the required set of genetic interactions *in silico*. Our model accounts for the expression of four mutually interacting gap genes, *hunchback *(*hb*), *Krüppel *(*Kr*), *giant *(*gt*), and *knirps *(*kni*), and takes into account the expression of the genes *bicoid *(*bcd*), *caudal *(*cad*), and *tailless *(*tll*) as external inputs. Parameter values in the model were calculated by fitting solutions to mean time dependent expression levels from zygotic and maternal/zygotic genes and *bcd *expression from a single embryo. The resulting model, when run with Bcd gradients from many individual embryos, correctly predicted the variance in position of six gap gene borders. Analysis of model behavior at the numerical level showed that the observed variance was a consequence of gap gene cross regulation [3]. To further elucidate the general nature of the mechanisms controlling variance, we turned off diffusion and analyzed the circuit in individual nuclei using ideas from dynamical systems theory.

This analysis showed that the observed reduction in variation of gap gene expression patterns is a consequence of the action of robust attracting states [4]. The formation of borders of gap gene expression domains could be understood in terms of three qualitative dynamical mechanisms: (1) The movement of attractors; (2) Selection of attractors; (3) Selection of states on a one dimensional attracting manifold. The last of the three mechanisms also causes the domain shifts of the gap genes.

There were two limitations to this analysis. First, the dynamical interactions underlying the observed reduction in variance were elucidated by performing the dynamical analysis on one particular circuit controlled by the median Bcd gradient used for the fit. As a consequence, the variance reduction analysis described in [4] was shifted towards considering only how attractors canalize the variance of initial conditions in the dynamical system, without characterization of Bcd dependence of these attractors. It is possible that other Bcd gradients may entail different dynamical mechanisms of pattern formation. A second limitation is that the individual Bcd gradients used in the analysis had certain systematic scaling errors that exaggerated the variation in threshold location [3,14]. In this paper we extend the dynamical analysis to multiple Bcd gradients, revealing additional dynamical mechanisms which nevertheless work in a coordinated manner to reduce variance. Moreover, we extend the analysis to a system in which the systematic exaggeration of Bcd variance has been removed.

The key idea of this extended analysis is to express gene expression variability in terms of such basic objects of the dynamical systems theory as attractors and attraction basins. The canalization will be explained by specific geometrical properties of these objects. We demonstrate the applicability of this approach using *hb *border formation as an example. We analyzed how this border forms in terms of the phase portrait of the gap gene dynamical system for various Bcd profiles. We find two specific mechanisms responsible for canalization of the variance, both connected to the phase portrait geometry.

## Methods

### The ensemble of Bcd concentration profiles

Two sets of spatial profiles of the Bcd concentration were obtained by the numerical processing of raw Bcd data from individual embryos in two different ways. One set was identical to that previously described [3]: the Bcd profiles were retrieved from 89 embryos in the FlyEx database [15-17], with the background removed by basic normalization as described [18]. This method as applied to Bcd profiles assumes that the Bcd profile is exponential and that the background profile is quadratic, a point independently supported by staining in null mutants. One embryo was rejected because of a nonexponential profile [3], thus in total 88 profiles were used in the study. For crosschecking purposes, we applied an alternative normalization method to the same raw Bcd data set as described [14]. The method adjusts both concentration scale and levels of constant background to minimize variance in the ensemble. This way of renormalizing data is useful because it is clear from comparison to *in vivo *work that our profiles exaggerate variance in Bcd amplitude. However, because there is no independent reason to believe that background should be adjusted to minimize variance, the *in situ *data processed in this manner should be viewed as a lower limit on embryo to embryo variation of the Bcd profile.

The normalized Bcd profiles were approximated by exponential functions *v*^Bcd^(*x*) = *A *exp(-*lx*) with *x *varying along the A-P axis of the embryo. We obtained in this way a set of 88 (89 for the alternative normalization method) Bcd parameters {*A*, *l*} (Additional file 1: Figure S1). The Bcd concentrations in different nuclei at the A-P axis were calculated as , where *x_i _*was the position of *i*th nucleus. We selected a "median" Bcd profile by picking an embryo with parameters {*A*, *l*} closest to the centroid point in the set of all {*A*, *l*} values. This median profile was used to fit the gap gene circuit as described [3].

### The gap gene circuit

We modeled the expression of the network of four gap genes *hb*, *Kr*, *gt*, and *kni *with the following equations [3,4,13,19]:(1)

where  is the concentration of protein encoded by gene *a *(1 ≤ *a *≤ *N *, *N *= 4) in nucleus *i *(1 ≤ *i *≤ *M*(*n*)) along the A-P axis of the embryo. The model incorporates nuclear divisions, number of nuclei *M*(*n*) and diffusion coefficients *D^a^*(*n*) depend on the cleavage cycle number *n*. The function  has a sigmoidal graph and describes regulated scaling for the maximal rate *R^a ^*synthesis of the *a*th protein. The argument *u *of *g *contains inputs from various transcriptional regulators. The first input is a linear combination of all  from the network with parameters *T^ab^*, which thus quantify the regulatory interactions between the genes. The Bcd concentration profile  is a time invariant maternal input to the system. The term  is a given time-dependent external input from the transcription factors Caudal (Cad) and Tailless (Tll). The function *χ*(*t*) equals one during interphase and zero during mitosis, accounting for the fact that synthesis shuts down during this period. Coefficient  equals zero if *i *= 1 (*i *= *M*) and one otherwise, preventing protein diffusion outside of the spatial domain. Coefficient *λ^a ^*is the rate of protein degradation, and constant *h^a ^*adjusts the threshold of the regulation function.

The initial conditions in the model consist of a spatial gradient of maternally expressed Hunchback (Hb) protein concentration and zero concentrations of the other three proteins. These conditions correspond to the state of the gap gene system at the start of cleavage cycle 13. The solutions in the model are biologically meaningful until the end of cleavage cycle 14A, at which time the midblastula transition occurs and many properties of the embryo change. The nuclei range along the A-P axis within the spatial domain from 35% to 92% of the embryo length (EL) where the gap genes express [3,4,13]. This spatial domain includes 30 nuclei in cleavage cycle 13 and 58 in cycle 14A.

All gene expression levels  are on a scale of 0-255 chosen to maximize dynamic range in the experimental data without saturation. These levels are called relative concentration units throughout the paper. Time dependent inputs for  and  were obtained from the data by averaging Cad and Tll expression patterns over individual embryos at various time points, as described in details in earlier work [2]. The background was preliminary removed from the individual Cad and Tll patterns as described [18]. The parameters for the model with Bcd normalized as described [18] were those reported in (See Supplementary Material in [3]). For the alternative normalization procedure, we selected a new median Bcd profile from the renormalized data and fitted the model with that profile to the same time dependent averaged gap gene data used previously by either serial or parallel Lam simulated annealing [11,20].

### Simplified equations

We considered a simplified version of the model without diffusion, called the "shorted model" as in previous work [4], keeping the parameter values equal to those found by the fitting procedure in the full model (1). Neglect of the diffusion term in (1) decouples nuclei and reduces 4 × *M *model equations to *M *independent systems of four equations, each system corresponding to one nucleus. We investigated only a restricted region of the A-P axis surrounding the posterior border of the anterior *hb *expression domain. The region ranges from 37% to 57% EL, about 11 nuclei in width in cleavage cycle 13 and 22 in cleavage cycle 14A. Tll does not act in this region and can be omitted [4]. We can then analyze shorted equations [4] given by(2)

where index *x *indicates the parametric dependence of solutions. It stems from the dependence of Bcd and Cad protein concentrations on spatial position as determined from experimental data. Such dependence can come directly from experimental data in nuclei, for which *x *= *i*, or as a continuous real-valued interpolation *x*.

The earliest observable indications of the mid-blastula transition, which include decay of the Bcd gradient, are evident after time class 6 (T6) of cycle 14A, about ten minutes prior to the onset of gastrulation [2,4]. For this reason, we represent the time dependence of *v*^Cad^(*x*, *t*) from the beginning of cleavage cycle 13 to the end of time class T6. Thereafter we take *v*^Cad^(*x*, *t*) = *v*^Cad^(*x*, T6) and *χ*(*t*) = 1 for times *t >*T6. Thus, the shorted equations (2) are nonautonomous until T6 and autonomous later.

### Attracting sets, basins of attraction, and hb border positions

We analyzed the dynamical system (2) for spatial position *x *from 37%-57%EL region in two mutually complementary directions. We performed a bifurcational analysis in the autonomous version of the system (2), in which *χ*(*t*) ≡ 1 and *v*^Cad^(*x*, *t*) was replaced by value *v*^Cad^(*x*, T6). The bifurcation structure in the system was studied on the Bcd-Cad plane, which is the plane with coordinates *v*^Bcd ^and *v*^Cad ^(with *v*^Cad ^corresponding to the Cad concentration at *t *= T6), by means of the AUTO package [21]. In this way, all equilibria and domains of their existence on the Bcd-Cad plane were calculated and all bifurcations separating these domains were elucidated. From another direction, at each spatial position of eleven nuclei in cycle 13 and for each of the 88 Bcd profiles (89 for the alternative normalization method), we calculated basins of attraction for each point attractor. This was done by evaluating the equations until late times when the solution is stabilized, with 10 000 random initial conditions uniformly distributed in the biologically relevant subspace of the initial conditions Ω = {0 ≤ *v*^Hb ^≤ 100, *v*^Kr ^= *v*^Gt ^= *v*^Kni ^= 0} (Ω is a part of the Hb axis in the 4D phase space of the dynamical system). Each point attractor is the asymptotic limit at late times of dynamics starting from the initial conditions which are grouped in a certain part of Ω, and we call this part the basin of attraction for the attractor. To get a more spatially refined picture, we also performed a calculation of attraction basins at thirty spatial positions in the range 37%-57%EL for the Bcd ensemble normalized by the basic method.

For each Bcd profile and fixed spatial position *x*, the attraction basin of each attractor can be represented as an interval (*c*_1_(*x*), *c*_2_(*x*)) on the Hb axis, as all other protein concentrations are zero in Ω. The values *v*^Hb ^= *c*_1 _and *v*^Hb ^= *c*_2 _are lower and upper boundaries for the basin, respectively. We used linear spatial interpolation for these values in order to study the attraction basin boundaries as continuous functions of *x *for each Bcd profile. Some attraction basins consist of more than one disjoint interval on the Hb axis. In this case, the basin boundaries comprise the boundaries of each connected part of the basin.

We calculated approximations for 1D unstable manifolds of saddles *S *having a single eigenvalue with positive real part by solving the simplified model equations from two initial conditions *S *± *w*, where *w *is a scaled eigenvector (corresponding to the unstable eigenvalue) of the Jacobian at the saddle.

In order to calculate the *hb *border positions in solutions exhibited at the onset of gastrulation, we used spatial interpolation of first and third order for solutions in the shorted and full models, respectively. The orders are different for the two models since the solution in the shorted model is less smooth in space than in the full one because of the absent diffusion term, and the use of high order interpolation schemes could lead to artifacts. As a consequence, we computed the border positions in different ways for the two models. The *hb *border position in the spatially interpolated solution of the shorted model was defined as the point in the spatial domain at which Hb concentration reached its half-maximal value, and in the full model as the local inflection point.

## Results

We extend the dynamical analysis reported previously for the median Bcd profile [4] to the entire ensemble of Bcd profiles first introduced in [3]. We placed exponential approximations of Bcd profiles from individual embryos in the model (2) with the parameter values calculated by Manu et al. (See Tables S1-S2 of Supplementary Information in [3]) and investigated characteristic features of the phase portraits in the model at various spatial positions.

**Figure 1:**
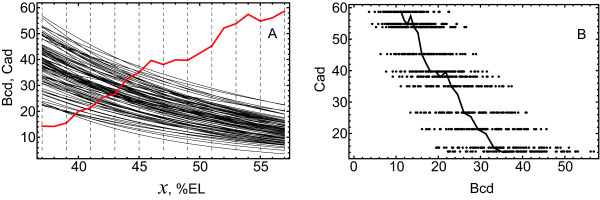
**Two representations of the spatial information in the model**. (A): The first representation is provided by the explicit use of the spatial position *x *at the A-P axis of the embryo. The panel shows the Cad spatial profile from time class 6 of cleavage cycle 14A (red line) and exponentially approximated individual Bcd profiles in the ensemble (black lines). The vertical dashed lines indicate positions of eleven nuclei in cleavage cycle 13 and in the given spatial range. (B): The second representation is provided by the use of 2D parameter (*v*^Bcd^, *v*^Cad^), specifying values of Bcd and Cad concentrations in Eqs. (2). This parameter defines a point on the Bcd-Cad plane. The dots in the panel represent the points whose Cad components come from the intersection points between the dashed lines and the Cad profile in (A), and the Bcd components from the intersections of dashed lines with the Bcd profiles in (A). Therefore, these dots describe the actual values of the external input (*v*^Bcd^, *v*^Cad^) in Eqs. (2) at late times and for the selected eleven spatial positions. The solid line in the panel is the curve  parameterized by *x *from the spatial range, where  is the median Bcd profile and *v*^Cad^(*x*) is the Cad profile from (A), showing how the variation of spatial position *x *in the model with the fixed Bcd profile is read on the Bcd-Cad plane.

In visualizing the results of our analysis, it is useful to consider two equivalent representations of the spatial information coded in Eqs. (2), either an explicit parameter *x *denoting A-P positions in the range 37%-57% EL or a point on the Bcd-Cad plane (Figure 1). We further use the Bcd-Cad plane to show the bifurcation diagrams and discrete spatial positions for presenting attraction basins.

**Figure 2:**
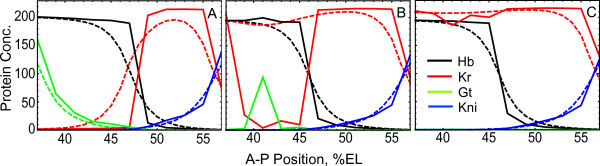
**Three classes of solutions**. Examples of solutions at time class 6 (*t *= T6) in the full (dashed lines) and simplified (solid lines) models for three different Bcd profiles corresponding to three classes I-III (A-C, correspondingly). The simplified model equations were obtained from the full model equations by neglecting the diffusion term and the influence from Tll (see Methods). (A): In class I, all borders are present and in the correct order, as is the case with the median Bcd profile. (B): In class II, the anterior *Kr *border is missing in the full model but present in the simplified model, typically in association with ectopic anterior expression of *Kr *in place of *gt*, possibly with some ectopic *gt *expression. (C): In class III, the anterior *Kr *border and anterior *gt *domains are absent in both the full and simplified models.

For the median Bcd profile, the gap gene expression patterns generated by Eqs. (2) from 35% to 71% EL occur in the same order and locations as those generated by the full model equations (1). The only exception is that the borders become very sharp and domains tend to be mutually exclusive (See Figure 2 in [4]). Over the full ensemble of Bcd profiles, we found three classes of behavior associated with the qualitatively different expression patterns of genes *Kr *and *gt *in the anterior vicinity (Figure 2). These classes were visible, but blurred by diffusion, in our previous study of the numerical behavior of the full model (See Figure 3A in [3]).

### Attracting sets and various mechanisms of border formation

**Figure 3:**
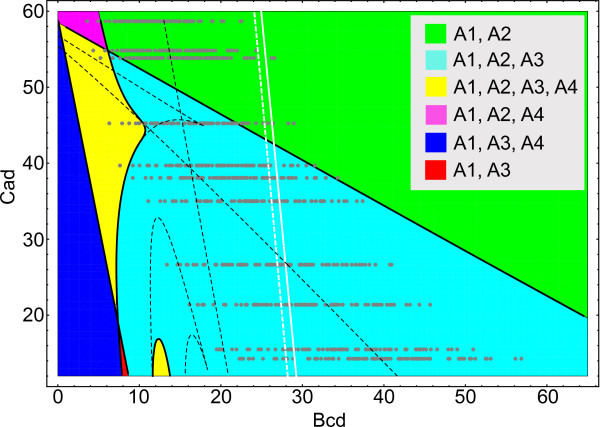
**The bifurcation diagram for the simplified model on the Bcd-Cad plane**. The abscissa axis shows *v*^Bcd ^values in the equations, and the ordinate axis is for *v*^Cad ^values corresponding to the Cad concentrations at times after T6, when the model equations are autonomous. The colored regions are domains of existence for point attractors *A*_1_-*A*_4_, described symbolically in Table 1. The boundaries (black solid lines) delimiting these domains represent the positions of all bifurcations affecting the attractors. The black dashed lines show the positions of bifurcations affecting only saddle equilibria. The white dashed and solid lines are loci of points where the Gt concentration at attractor *A*_1 _equals 50 and 150, respectively. Therefore,  to the left of the white dashed line,  to the right of the white solid line, and  between the lines. The gray dots are the same as in Fig. 1B.

For each Bcd gradient, we performed an analysis of how border formation was driven by dynamical attractors. Because different Bcd profiles lie on different portions of the Bcd-Cad plane, we first characterized which combinations of attractors are present in different parts of this plane by performing a bifurcational analysis (Figure 3) of the shorted model (2). We then calculated the basins of attraction for all attractors at a discrete set of eleven positions corresponding to cycle 13 nuclei in the Bcd-Cad plane with initial conditions varied in Ω = {0 ≤ *v*^Hb ^≤ 100, *v*^Kr ^= *v*^Gt ^= *v*^Kni ^= 0}.

There are four stationary attractors (*A*_1_-*A*_4_) in the portion of the Bcd-Cad plane shown in Figure 3. The attractors can be coded with quadruples consisting of 0, X, or 1 for each attractor component by inspecting whether the corresponding protein concentrations have small, intermediate, or large values at the attractors (Table 1; see the existence domains for attractors corresponding to the alternative normalization method in Additional file 2: Figure S2; examples of spatial dependence of attractors are shown in Additional files 3 and 4: Figures S3 and S4 for the two normalization methods). For example, *A*_3 _= 0100 means that *Kr *is highly expressed at this attractor with the other genes staying repressed. *A*_1 _is the only attractor that continuously changes its code with varying Bcd and Cad concentrations. There are six domains on the Bcd-Cad plane in which various combinations of *A*_1_-*A*_4 _exist (Figure 3). Attractor *A*_1_ exists inside the entire portion of the considered plane, while the other attractors are involved in the bifurcations at the borders of their existence domains. There are three types of bifurcations found affecting attractors: saddle-node, Hopf, and Bogdanov-Takens bifurcations (see more detailed description of all bifurcations in Additional file 5: Protocol S1 and Additional file 6: Figure S5). A limit cycle appears at the Hopf bifurcation, which is a nonstationary oscillating attracting state. However, we have not found any oscillatory attractors when the initial conditions are varied in Ω, and, therefore, we exclude this type of attractor from the analysis. The gray dots in Figure 3 show the late time values of the external input in Eqs. (2) used in the calculations of attraction basins for eleven nuclei positions and all of the Bcd profiles. The location of the grey dots on the bifurcation diagram indicates the existence of specific attractors in the phase space for a given nucleus. However, not all of these attractors can be reached under the given biological initial conditions in Ω, as attraction basins of some attractors are disjoint with Ω (Additional file 7: Figure S6).

**Table 1:** The symbolic codes for all attractors in the study calculated for the Bcd and Cad ranges from Figure 3.

	*hb*	*Kr*	*gt*	*kni*
	1	0	0	0

	1	0	X	0

	1	0	1	0

*A*_2_	1	1	0	0

*A*_3_	0	1	0	0

*A*_4_	0	0	0	0

*A*_5_	0	1	0	1

*A*_6_	1	0	0	0

A component of an attractor is assigned the code value '1' if the concentration of the corresponding protein is larger than 150 relative concentration units, value '0' if it is smaller than 50 relative units, and value 'X' if it is between 50 and 150 (see Methods for description of relative units for protein concentrations). All attractors except *A*_1 _preserve a single code across the considered ranges for Bcd and Cad concentrations. Attractor *A*_1 _changes its state from  through  to  due to continuous increase of the Gt concentration for this attractor on the Bcd-Cad plane (see the existence domains for these states in Fig. 3 for the basic normalization method and Additional file 2: Figure S2 for the alternative one). All listed attractors exist in the model corresponding to the alternative normalization method, and only *A*_1_-*A*_4 _in the model associated with the basic method.

**Figure 4:**
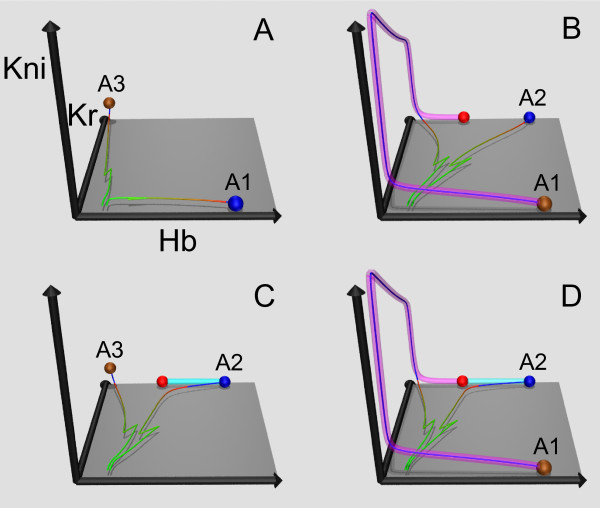
**Dynamical mechanisms of hb border formation for various Bcd profiles**. Each panel shows the Hb-Kr-Kni projections of the 4D phase portrait fragments from the *hb*-expressing and *hb*-nonexpressing border nuclei simultaneously. The *hb*-expressing and *hb*-nonexpressing border nuclei are the ones placed just anterior and just posterior to the *hb *border position, respectively. The axes labels for all panels are as for (A) and show the corresponding protein concentrations. The blue balls are the attractors from the *hb*-expressing border nucleus phase portraits, and the brown ones from the *hb*-nonexpressing nucleus portraits. The red balls are the saddles from the nuclei specified below for each panel. The cyan tubes are branches of the unstable manifolds for the *hb*-expressing border nuclei saddles, and the magenta ones for the *hb*-nonexpressing nuclei saddles. The green trajectories are the solutions for the biological initial conditions from the *hb*-expressing and *hb*-nonexpressing border nuclei. The initial conditions consist of the maternal Hb concentration  and are zero for all other proteins. The trajectories gradually turn to red as time approaches the end of cleavage cycle 14A (*t *= *τ*) and become blue for later times. (A): The picture is for Bcd profile #1, with the *hb*-expressing border nucleus at 49%EL  and the *hb*-nonexpressing border nucleus at 51%EL . (B): Bcd profile #71; the border nuclei at 49%EL  and 51%EL ; the red saddle is from the *hb*-nonexpressing border nucleus. (C): Bcd profile #6; the border nuclei at 47%EL  and 49%EL ; the red saddle is from the *hb*-expressing border nucleus. (D): Bcd profile #37; the border nuclei at 51%EL  and 53%EL ; the red saddle is from the *hb*-expressing border nucleus.

As shown elsewhere [4], the dynamics in the model for the median Bcd profile is qualitatively different in parts of the A-P axis which are anterior and posterior to the position of the bifurcation annihilating *A*_3_^. ^This position appears as a diagonal line running from the upper left to lower right of Figure 3. The anterior and posterior dynamical regimes are characterized by different types of attracting sets governing the solution dynamics. Solutions at the end of cycle 14A (*t *= *τ*) are very close to point attractors in the anterior regime and to attracting manifolds in the posterior one (examples of these attracting sets are shown in Figure 4). We found that these dynamical regimes are preserved across all the individual Bcd profiles.

To study the dynamical mechanisms of *hb *border formation, we examined the phase portraits in the shorted model at spatial positions on either side of the border. The mechanism determining the border was found by inspecting which attracting sets govern the solution dynamics from the biological initial conditions in the two nuclei, a *hb*-expressing border nucleus placed just anterior to the *hb *border position and a *hb*-nonexpressing border nucleus just posterior to that. Border formation is interpreted in these terms as a switch of solution between attracting states in these two nuclei.

We found four qualitatively different mechanisms of *hb *border formation for Bcd profiles from the ensemble, depending on the type of attracting sets approached by the solutions in the two nuclei by *t *= *τ *(Figure 4). For example, Figure 4B shows that for corresponding Bcd profile the border forms by a solution switching from attractor *A*_2 _= 1100 in the *hb*-expressing border nucleus to a *hb*-OFF state at the unstable manifold of one of the saddles in the *hb*-nonexpressing border nucleus. Other panels in the figure can be interpreted in a similar way.

There are 66 Bcd profiles associated with the phase portraits which exhibit the attractor-attractor switch mechanism of border formation (Figure 4A) and 20 profiles corresponding to the attractor-manifold switch mechanism (Figure 4B), which make these two mechanisms predominant for the Bcd ensemble. The other mechanisms shown in Figure 4C, D are rare and occur for only one Bcd profile each. The distribution of the four mechanisms over the three solution classes from Figure 2 is summarized in Additional file 8: Table S1. For all Bcd profiles, only attractors *A*_1_, *A*_2_, and *A*_3 _participate in *hb *border formation. *A*_4 _contains a nonempty attraction basin in Ω for only a few nuclei which lie in a portion of the Bcd-Cad plane that never contains the Hb border (Additional file 7: Figure S6).

### Spatial configuration of attraction basins

**Figure 5:**
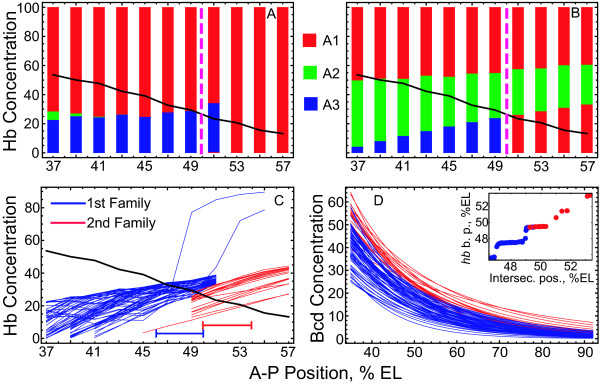
**Spatial configuration of attraction basins in the simplified model**. (A,B): Basins of attraction at the eleven spatial positions (nuclei in cleavage cycle 13) for the two Bcd profiles from the ensemble (profiles (A) #1 and (B) #71, corresponding to the phase portraits from Fig. 4A and 4B, respectively), together with the initial Hb concentration profile (black curve) and highlighted positions of the *hb *borders in the model (dashed magenta lines). Each thick vertical line presents the domain Ω, which is the set of initial values 0 *< v*^Hb ^*<*100 at the Hb axis, in which the attraction basins for attractors *A*_1_-*A*_3 _are marked by corresponding colors. The basins were calculated and shown at spatial positions ranging from 37% EL to 57% EL with 2% bins, and the width of the vertical lines containing these basins is arbitrarily chosen for better visibility. (C): Intersection of the initial Hb profile (black curve) and the attraction basin boundaries (blue and red curves) for all but one Bcd profiles classified in the two families in (D), as described in the text. The blue and red colors in both (C) and (D) correspond to the first and second Bcd families, respectively. For the first Bcd family, the initial Hb profile crosses a boundary separating either the attraction basins of *A*_1 _and *A*_3 _(see (A) for an example) or the basins of *A*_2 _and *A*_3_, and all these boundaries are shown as blue curves in (C) (see Methods for description of basin boundary calculation). For all Bcd profiles from the second family, the basin boundaries shown in (C) as red curves separate the basin of *A*_2 _and the lower part of the basin of *A*_1_ at various spatial positions (see (B) for an example). The two line segments at the bottom of (C) show the ranges of the *hb *border positions in solutions of the simplified model equations for Bcd profiles from the two families. The inset in (D) shows the correlation between the *hb *border positions in the model (vertical axis) and the positions of intersection points between the initial Hb profile and the basin boundaries from (C) (horizontal axis), for Bcd profiles from the two families.

To further investigate how the border formation mechanisms imply the observed *hb *border variance, we calculated the spatial and Bcd dependence of attraction basin boundaries. We visualize the way that the biological initial conditions choose a basin in different nuclei and try to connect it to the appearance of the *hb *border in the solution by the time *t *= *τ*. We construct graphs combining the initial Hb profile and the attraction basins for all attractors at different spatial positions, and highlight the range of positional variance for *hb*. Typical examples of these graphs are shown in Figure 5A,B.

For all but one of the Bcd profiles, *hb *border formation is associated with the transition of the initial Hb profile from the basin of one point attractor to the basin of another point attractor. This can be seen by comparing the attraction basins of the initial Hb concentration values in the two nuclei surrounding the Hb boundary position. For example, the transition in Figure 5A is from the basin of *A*_1 _in the *hb*-expressing border nucleus to the basin of *A*_3 _in the *hb*-nonexpressing border nucleus (the *A*_1 _→ *A*_3 _transition); the transition in Figure 5B is from the basin of *A*_2 _to the basin of *A*_1 _(*A*_2 _→ *A*_1 _transition). The basin-to-basin transitions can also be detected in Figure 4, where the solutions in the two nuclei go to different point attractors. Bcd profile #54 provides the only exception from this basin-to-basin transition rule. For this Bcd profile, the solution trajectories in both *hb*-expressing and *hb*-nonexpressing border nuclei eventually end up at the same point attractor *A*_2_. The *hb *border forms in this case according to the mechanism from Figure 4B (the attractor-manifold switch) with the only difference that the solution trajectory in the *hb*-nonexpressing border nucleus is attracted to a *hb*-OFF state on an unstable manifold which connects a saddle and attractor *A*_2 _(data not shown). This Bcd profile was excluded from the further analysis. For all cases with the *A*_1 _→ *A*_3 _transition, attractor *A*_1 _is in the state  for the *hb*-expressing border nucleus. The *A*_1 _→ *A*_3 _and *A*_2 _→ *A*_3 _transitions are similar in the sense that they both correspond to the switch from an attractor with *hb*-ON state to an attractor with *hb*-OFF state. We found 69 Bcd profiles in the ensemble associated with either of these two transitions. We call these profiles Family I in what follows. These transitions can hypothetically correspond to any of the four mechanisms possible for the *hb *border formation, because the attracting invariant manifolds may also participate in the border formation in this case (see, e.g., Figure 4C). On the other hand, the *A*_2 _→ *A*_1 _transition from Figure 5B describes the transition of the initial Hb concentration between the basins of attractors which both have the *hb *component in an 'ON' state. The *hb *border forms in this case by the attracting manifold which provides the necessary *hb*-OFF state at *t *= *τ *(see Figure 4B,D). We found a total of 18 Bcd profiles leading to the *A*_2 _→ *A*_1 _transition. We refer to these profiles as Family II. Among these profiles, 16 are associated with the state  and 2 profiles with the state  for the *hb*-nonexpressing border nucleus. The basin of *A*_3 _in Figure 5A stops to exist for nuclei located posterior to 51%EL. The system is in the basin of *A*_1 _for these positions and approaches a *hb*-OFF state by *t *= *τ *with the help of an attracting manifold. This participation of an attracting manifold in attaining a *hb*-OFF state is the distinctive feature of the aforementioned posterior dynamical regime.

Table 2 shows how the types of initial Hb transitions between basins are distributed with respect to the border formation mechanisms described above.

**Table 2:** The distribution of Bcd profiles over the four mechanisms of hb border formation and two families described in the text.

	AA	AM	MA	MM
1st family	66	2	1	0

2nd family	0	17	0	1

The mechanisms are labeled as follows: attractor-attractor switch (AA; Fig. 4A), attractor-manifold switch (AM; Fig. 4B), manifold-attractor switch (MA; Fig. 4C), and manifold-manifold switch (MM; Fig. 4D).

### Variability of basin boundaries and hb border positions

The fact that the *hb *border forms according to the basin-to-basin transition of the initial Hb profile implies that this profile crosses a boundary between two basins. Therefore, we can relate the border position to the position of the intersection point between the initial Hb profile and the spatial profile of the basin boundary (see Methods for a description of how this profile is calculated). One can speculate that these two positions are the manifestation of two levels of positional information readout in the gap gene circuit. In this subsection, we compare the positional variability for *hb *predicted by the model for these two levels. We calculated the spatial profiles of the basin boundaries for all Bcd gradients. The ranges of the border positions corresponding to the two Bcd families are shifted with respect to each other, and the same shift is observed at the level of the basin boundaries (Figure 5C,D). As expected, the positions of the *hb *border and basin boundary intersection points exhibit strong correlation for both Bcd families (see the inset in Figure 5D). Note that the step-like form of the correlation curve in the figure follows from two factors, the discrete spatial positions used in calculations and the absence of diffusion in the model equations (i.e., the solution of the simplified model more sharply depends on the spatial position).

This correlation means that the intersections between the initial Hb profile and specific basin boundaries encode the *hb *border positions. As shown in Table 3, the canalization of Bcd variation takes place both at the level of the basin boundaries and at the level of *hb *border in the simplified model. The variabilities of the intersection points and the *hb *border positions are approximately the same for each Bcd family and between the families. On the other hand, the Bcd positional variance is significantly different in the two families.

**Table 3:** Positional variance for Hb and Bcd.

	First Bcd family		Second Bcd family		Full Bcd set	
	
	**f.r**.	**s.d**.	**f.r**.	**s.d**.	**f.r**.	**s.d**.
Intersection positions	2.9	1.0	4.0	1.2	6.6	1.5

*hb *border positions	3.9	1.4	4.0	1.3	7.9	1.8

Bcd threshold positions	13.2	3.6	6.1	1.8	19.7	4.5

Variability of the *hb *border positions in the simplified model and the intersection points between the initial Hb profile and the attraction basin boundaries (from Fig. 5C) for two Bcd families and for the whole Bcd set, in comparison with the positional variability of threshold Bcd concentration. The Bcd threshold value for a family was chosen by picking a Bcd profile corresponding to the mean position of the *hb *border for that family; then, the threshold value was equal to the value of this Bcd profile at that position. The full range (f.r.) and standard deviations (s.d.) are given in % of embryo length. The standard deviation for the *hb *border is 1.3% in solutions of the full model and 1.0% in data [3].

### A nonlinear curve of hb border response to Bcd variation

**Figure 6:**
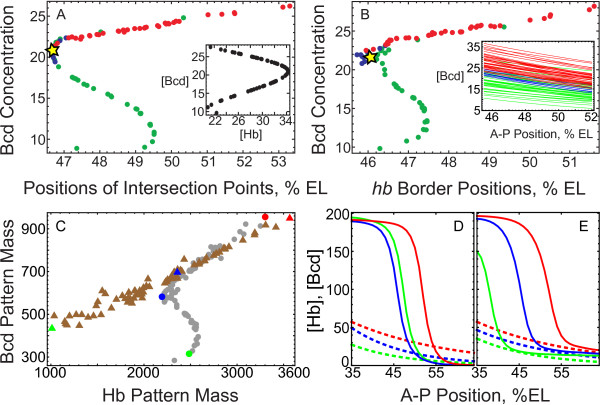
**The nonlinear curve of hb border response to Bcd variation in (A) the simplified and (B-E) full models**. (A): The value of Bcd concentration at the A-P position where the initial Hb profile intersects the corresponding basin boundary from Fig. 5C versus this position, for all but one Bcd profiles from the ensemble (Bcd profile #54 was excluded from the analysis as explained in the text). The colors mark the three solution clusters I-III from Fig. 2: green corresponds to class I, blue to II, and red to III. The yellow stars indicates the median Bcd case. The inset in (A) shows the Bcd dependence of the attraction basin boundary for the attractor *A*_3 _at fixed A-P position equal to 47% EL. As explained in Methods, the attraction basin has the form of interval (*c*_1_,*c*_2_) at the Hb axis; the Bcd concentration at 47% EL versus the value [Hb] = *c*_2_ at the same position is plotted in the inset for all Bcd profiles from the first family. (B): The same response curve as in (A) but obtained from calculations in the full model (1) and with the *hb *border positions used instead of the intersection point positions. The inset shows all Bcd profiles used in the calculations. Colors in (B) correspond to those in (A). (C): The response curve expressed in terms of 'masses' of the Bcd patterns and Hb solutions. The mass is calculated as the integral of a pattern (a Bcd profile or a Hb solution at the end of cleavage cycle 14A) over the spatial region 35-64%EL. The Hb pattern mass positively correlates with the *hb *border position (data not shown). The dots represent results of calculations in the full model, while the triangles correspond to calculations with the same equations but with the gap gene cross regulation of *hb *turned off (*T*^*hb*←*a *^set to zero in (1) for each gap gene *a*). The green, blue, and red symbols indicate cases with a small, intermediate, and large mass of the Bcd patterns, respectively. The Bcd and Hb patterns used in calculations of the colored dots are shown in (D) with respective colors, and the same for triangles in (E). The Bcd patterns are dashed lines, and the Hb solutions are solid ones.

The Bcd dependence of *hb *border can also be investigated by inspecting the curve representing the response of *hb *border position to Bcd concentration levels in a vicinity of this position. We studied this response prescribed by the model for Bcd profiles from the ensemble (Figure 6).

The response curve exhibits a specific nonlinear form which is the same in the simplified model (Figure 6A) and in the full one (Figure 6B-C). It persists even if we recalculate the curve by using the normalized individual Bcd profiles instead of their exponential approximations (Additional file 9: Figure S7), indicating that the results are independent of the nuclear noise present in the non-approximated Bcd data.

The nonlinear form of the curve is the result of the reaction to Bcd variation from the whole gap gene network. As a consequence, we have a linearly increasing response curve when gap gene cross regulation of *hb *is removed from the full model equations (Figure 6C). In that case, the gradually increasing amplitude (or a mass) of Bcd profile across the ensemble results in gradual posterior shift of the *hb *border (Figure 6E). This happens because Bcd is an activator of *hb *and the spatial profiles of both Bcd and Hb concentrations are monotonously decreasing in the vicinity of *hb *border position. In contrast, there can be reversed (anterior) shifts of the *hb *border for some range of Bcd concentration in the presence of gap gene cross regulation (Figure 6D).

The inset in Figure 6A shows an example for one spatial position of how the attraction basin boundary depends on Bcd concentration. This dependence locally represents the response curve at the level of attraction basins in the model. The specific form of the curve in the inset underlies the nonlinear form of the response curve for the intersection points and border positions.

### Results for the alternative Bcd normalization method

As the Bcd variance can be exaggerated in the data normalized by the basic method, we considered the same ensemble of Bcd profiles but normalized by the alternative artificial normalization method, which minimizes variance in the ensemble (see Methods). We studied the model on the newly normalized Bcd profiles in order to crosscheck our results. The corresponding parameters *A *and *l *of exponential approximation for the Bcd profiles are shown in Additional file 1: Figure S1. We calculated a new set of parameter values (Additional file 10: Table S2) in the full model (1) with new median Bcd profile by fitting to the same gap gene expression data that were used previously [3,4]. Main qualitative features of these parameter values and corresponding solution of the simplified equations (2) (Additional file 11: Figure S8) are very close to those in the above study [3,4].

Due to the specific statement of the alternative normalization method, the Bcd positional variance in the ensemble became much lower: the standard deviation *σ *= 1.2%EL. The corresponding positional variance for *hb *in the full model equals to *σ *= 0.5%EL.

We calculated a new bifurcation diagram on the Bcd-Cad plane for the simplified model (Additional files 2, 12, 13: Figures S2, S9, and Protocol S2). There are two new attractors, *A*_5 _= 0101 and *A*_6 _= 1000, in addition to attractors *A*_1_-*A*_4 _which are analogs of the corresponding attractors to the above study. Attractor *A*_6 _exists in a very narrow domain on the Bcd-Cad plane, where it coexists with attractor *A*_1 _at the state  (Additional file 2: Figure S2). Thus, the attractor 1000 is unique in this domain. The calculations of attraction basins at discrete spatial positions did not detect *A*_4_, since the newly normalized Bcd concentrations never entered the existence domain for this attractor (Additional file 14: Figure S10). As in the above study, only attractors *A*_1_-*A*_3 _are important for the process of *hb *border formation. The spatial dependence of these attractors is mostly similar to the previous results, except that the bifurcations changed their positions at the A-P axis (see, e.g., the case of the median Bcd profile in Additional file 4: Figure S4, in comparison with previously discussed Additional file 3: Figure S3). Despite the difference in the bifurcation structure, new calculations also revealed the existence of an attracting invariant manifold governing the dynamics in a posterior spatial region, which is the analog of the previously found manifold [4]. This manifold has similar geometrical properties, reflecting the fact that it is responsible for the observed anterior shifts of the posterior gap domains taking place in cycle 14A [4].

Three of the four mechanisms shown in Figure 4 exist for the new parameter values and for various newly normalized Bcd profiles: the attractor-attractor (Figure 4A), manifold-attractor (Figure 4C), and manifold-manifold (Figure 4D) switches. Analyzing how the initial Hb profile chooses the attraction basin in the nuclei neighboring the *hb *border, we detected 85 Bcd profiles leading to the *A*_1 _→ *A*_3 _transition, 2 Bcd profiles leading to the *A*_2 _→ *A*_3 _transition, and 2 profiles which were not associated with the change of basins across these nuclei (they corresponded to the switch between different states on the unstable manifold of a saddle) (see Additional file 15: Table S3 for a distribution of these cases with respect to the solution classes I-III). The model demonstrates a picture of the initial Hb profile crossing the attraction basin boundaries similar to the first family case in Figure 5C, but with a smaller variance (Additional file 16: Figure S11). The Bcd response curve for the *hb *border in the model with the new parameter values is also not linear (Additional file 17: Figure S12).

## Discussion

### Mechanisms of border formation

We presented the dynamical analysis of the simplified model of the gap gene network on the ensemble of early *Drosophila *embryos. The main goal was to decode the mechanistic basis of the gap gene border formation and stability under the Bcd morphogen variance. The *hb *border formation mechanisms were described in terms of attracting sets and their attraction basins calculated in the nuclei surrounding the border position.

The results reveal that the border formation can be associated with the event of intersection between a boundary separating the attraction basins of two different point attractors and the initial Hb profile presenting the input from the maternally expressed *hb *gene. Attracting sets of another type, the unstable manifolds of saddle equilibria, actively participate in the adjustment of the border position. They do so by attracting the solution trajectories in the nuclei surrounding this position. The model predicts that these attracting manifolds can be involved in the border formation for some Bcd profiles.

The *hb *border correctly forms in the model by the onset of gastrulation for all individual Bcd profiles. For about a half of these profiles, however, the Kr and Gt patterns in the solutions exhibit defects in the anterior part of the spatial domain (solution classes II and III). It turns out that the *hb *border formation mechanism involving the attracting manifolds is mostly associated with these cases. This may lead to the conclusion about restricted applicability of this mechanism in the case of *hb *expression. However, this mechanism exists and plays an important role for the gap domain borders in a posterior part of the embryo, where the domains form and vary in time under the control of an unstable manifold [4]. To analyze canalization for the posterior borders, the variation for external inputs from Cad and Tll should be taken into account, where these transcription factors are among the key regulators, and a modified model should be considered including an input from the terminal gene *huckebein *[22].

### Mechanisms of canalization

As previously reported, the model exhibits a significant filtration (canalization) of the Bcd positional variability at the level of *hb *border formation [2-4]. Our results show how this filtration stems from the stable behavior of the attraction basin boundaries. It was shown in [3,10] that the mutual regulatory repression between the gap genes accounts for the observed variance reduction, thus presenting a buffering mechanism for canalization [23,24]. We translated this buffering mechanism to the level of attractors and their attraction basins. As the *hb *border position is well encoded by the intersection between the initial Hb profile and corresponding attraction basin boundaries, the stability of *hb *border predicted by the model can be explained by inspecting the geometrical properties of these attraction basins.

From this inspection, we can elucidate the following two mechanisms responsible for the observed robustness. First, the initial Hb profile is a monotonously decreasing function of A-P position, while the basin boundary to be crossed is a monotonously increasing one (Figure 5), i.e., these curves have opposite dependencies on the A-P position. This *purely geometrical *fact evidently prescribes a smaller variation of the intersection point when the basin boundary is changing due to the variance of Bcd concentration, as opposed to the case if the curves would jointly rise or jointly fall along the A-P axis (we illustrated this mechanism of canalization in Additional file 18: Figure S13).

The second mechanism is associated with the specific nonlinear form of the response curve from Figure 6. The gap gene cross regulation of *hb *bends the response line exhibited in absence of this regulation (Figure 6C). This bending effectively reduces the Hb positional variance by about half. In terms of attractors, this bending is controlled by the fact that a basin boundary responsible for the *hb *border formation does not change monotonously, but oscillate in the state space with the changing Bcd profile.

The results show that the full range of the *hb *positional variance is broken down into two almost equal parts, the anterior and posterior ones (see the line segment in Figure 5C). These parts are associated with two families of the Bcd individual profiles (Family I and Family II, respectively) and two different mechanisms of *hb *border formation. The Bcd profiles from Family I lead to the *hb *border formation as a switch from a *hb*-ON attractor in a *hb*-expressing nucleus to a *hb*-OFF attractor in a *hb*-nonexpressing nucleus, while for Family II the border forms with the help of an attracting invariant manifold in a *hb*-nonexpressing nucleus. Since the difference between the two families is in the amplitude of the Bcd profiles, we conclude that Bcd profiles of high amplitude canalize by a dynamical mechanism different from those of lower amplitude. Each dynamical mechanism provides only half of the full variance for the *hb *border, but in two adjacent spatial domains. Therefore, the change of the dynamical mechanism that happens with rising Bcd amplitude effectively doubles the variance.

The *hb *border positions from the more posterior range are placed posterior to the spatial position of a bifurcation annihilating attractor *A*_3_. This bifurcation position delimits the anterior and posterior dynamical regimes in the model, as described previously [4]. Therefore, the Bcd profiles from the second family shift the *hb *border to the posterior dynamical regime, which is characterized by an active role of an attracting invariant manifold in the pattern formation.

The results indicate that the posterior range of *hb *positional variation is almost equal to the anterior one only due to smaller variation of the Bcd profiles in Family II compared to Family I. This suggests that the solutions in the anterior and posterior dynamical regimes have quite different sensitivity rates to variation of the Bcd concentration. For Family I, the standard deviation for the *hb *border position is 2.6 times less than for the Bcd threshold position, while it is only 1.4 times less in the case of Family II. This difference can be explained by an observation that Bcd profiles of higher amplitude correspond to the linear part of the response curve from Figure 6, and this is a consequence of specific regulatory interactions in the gap gene circuit as explained further.

We have used the model (1) to study the canalization mechanisms based on the assessment that the model provides one of the best spatio-temporal precision for the description of gap gene expression [25]. This model is an approximation to a more general model of gene regulation, which should be grounded on the statistical-mechanical formalism. One possible limitation is the linear approximation for the argument of the nonlinear regulation function *g*. The canalization mechanisms described in terms of attractors and attraction basins generally depend on the structure of the model that predicts these attracting states. Therefore, an important direction for future investigations should be verification of the proposed mechanisms in a phase space of a more general model.

### Response curve

The nonlinear nature of the Bcd readout by the gap gene circuit is clearly represented in a specific nonlinear form of the response curve showing the Bcd dependence of the *hb *border position in the model. The nonlinear part of the curve can be explained by the regulatory actions on *hb *from the other gap genes. In particular, a regulatory analysis in the full model revealed that the regulatory interactions between *hb*, *gt*, and *Kr *underlie the folding part of the response curve (Additional file 19: Protocol S3). The gap gene cross-regulation also participate in the linear parts of the response curve by tuning the incline of these parts.

It was previously pointed out that the *gt *and *Kr *expression borders in the anterior part of the A-P axis show large variation in the model in response to Bcd variation because the model is missing some regulators in this part [3]. For example, these *gt *and *Kr *borders are absent in the solutions from class III. This fact raises doubts on the specific folding part that the response curve exhibits in the middle range of the Bcd concentration values. On the other hand, the folding part exists only for the Bcd profiles associated with the solutions from class I, with all expression borders formed correctly, which means that an essential portion of the artificial variation of the *gt *and *Kr *borders can be excluded from the consideration without affecting the folding form of the curve.

### Comparison of results for two normalization methods

We investigated the model on the ensemble of Bcd profiles normalized by the alternative method, which provided lower Bcd variance [14]. One used this method as an artificial limit case, in which we dealt with the ensemble possessing minimal Bcd variance, and applied it for the crosschecking purposes.

We have not found any essential discrepancy in the mechanisms of *hb *border formation and canalization for the two normalization methods. A distinct bifurcation structure in the model with the new parameter values does not lead to changes in the solutions during the biologically important time. The model preserves an attracting invariant manifold related to the posterior dynamical regime. The same border formation mechanisms appear except the one associated with the attractor-manifold transition. It is important that, even though the second family of Bcd profiles does not appear in the alternative normalization case, the invariant manifolds still play their role in adjusting the border position. The model also demonstrates an essentially nonlinear response curve for the *hb *border. Therefore, our conclusions formulated above are robust with respect to the choice of the normalization method, and, in more general terms, they should be valid for different estimates of the actual Bcd variance.

This correspondence can be explained by the fact that the parameters *A *and *l *obtained for the alternatively normalized Bcd profiles form a subset in similar parameters obtained in the case of the basic normalization method (see Additional file 1: Figure S1). Roughly speaking, we can associate the alternatively normalized Bcd profiles with Family I. In particular, this means that the Bcd data rescaled according to the alternative algorithm support the conclusion formulated above about different dynamical mechanisms of canalization for Bcd profiles of different amplitude.

There is an important issue concerning the comparison of the Bcd variance filtration rates. The calculations reveal that, for the basic normalization method, the Hb positional variation of 1.3%EL in the model output follows from the Bcd positional variation of 4.5%EL, thus implying that more than 70% of the positional variance has been filtrated. The same calculations for the alternative normalization method give the filtration rate of approximately 60%. Therefore, the filtration still happens in the model even if we normalize Bcd profiles according to the precisionist hypothesis [14]. This result is quite expected since the reported dynamical mechanisms underlying the processing of the Bcd variation in the model are valid irrespective of the absolute variation range. Whatever actual variation the Bcd morphogen exhibits, the nonlinear model response translates it to a smaller variation of the target gene patterns.

## Conclusions

The formation of *hb *border is coded by the intersection between the maternal Hb gradient and a boundary between attraction basins in the gap gene dynamical system. Small positional variance for *hb *border can be explained by the geometrical properties of this basin boundary and its nonmonotonic dependence on the Bcd concentration. Main features of the phase portraits underlying the canalization mechanisms do not depend on the normalization method for Bcd.

## Authors' contributions

VVG, M, and JR conceived and designed the course of the study. VVG performed calculations of attractors and attraction basins, model optimization, and analysis of data and results. LP carried out the bifurcational analysis and calculated the phase portraits. EMM applied alternative normalization to the Bcd data and analyzed the data. MGS and JR performed biological analysis of the results. VVG and LP made the figures. AMS, MGS, and JR supervised the work. VVG, LP, JR, MGS, and AMS wrote the paper. All authors read and approved the final manuscript.

## Supplementary Material

Additional file 1 **Parameters *A *and *l *of exponential approximation of individual Bcd profiles for two normalization methods**. Click here for file

Additional file 2 **The bifurcation diagram for the new parameter values**. Click here for file

Additional file 3 **The spatial dependence of attractors in the shorted model for the median Bcd profile**. Click here for file

Additional file 4 **The spatial dependence of attractors in the shorted model with the new parameter values and for the median Bcd profile**. Click here for file

Additional file 5 **Detailed description of the bifurcations in the model (the text contains reference to Figure S5)**. Click here for file

Additional file 6 **The bifurcation diagram with more details**. Click here for file

Additional file 7 **The existence domains on the Bcd-Cad plane for attractors *A*_1_-*A*_4 _following from calculations at discrete spatial positions**. Click here for file

Additional file 8 **The distribution of Bcd profiles over the four mechanisms of *hb *border formation and over solution classes I-III**. Click here for file

Additional file 9 **The response curve for the normalized individual Bcd profiles instead of their exponential approximations**. Click here for file

Additional file 10 **New parameter values in the model obtained by optimization with a median Bcd profile from the Bcd data normalized by the alternative method**. Click here for file

Additional file 11 **The solutions of the full model equations and their simplified version for the new parameter values**. Click here for file

Additional file 12 **The bifurcation diagram for the new parameter values with more details**. Click here for file

Additional file 13 **Detailed description of the bifurcations for the new parameter values (the text contains reference to Figure S9)**. Click here for file

Additional file 14 **The existence domains on the Bcd-Cad plane for attractors *A*_1_-*A*_6 _in the model with the new parameter values following from calculations at eleven spatial positions**. Click here for file

Additional file 15 **Classification results for the Bcd profiles in the case of the alternative normalization method**. Click here for file

Additional file 16 **The spatial configuration of attraction basins in the model with the new parameter values**. Click here for file

Additional file 17 **The response curve for the new parameter values**. Click here for file

Additional file 18 **Schematic illustration of the first canalization mechanism**. Click here for file

Additional file 19 **The regulatory analysis of the response curve (the Protocol contains Figures S14-S17)**. Click here for file
