# Supplementary material for: Mechanisms of gap gene expression canalization in the Drosophila blastoderm
Source: BMC Syst Biol. 2011 Jul 28;5:118. doi: 10.1186/1752-0509-5-118 (PMC3398401; doi:10.1186/1752-0509-5-118)
Supplement: Additional file 1 — Parameters A and l of exponential approximation of individual Bcd profiles for two normalization methods. [file 1752-0509-5-118-S1.PDF]

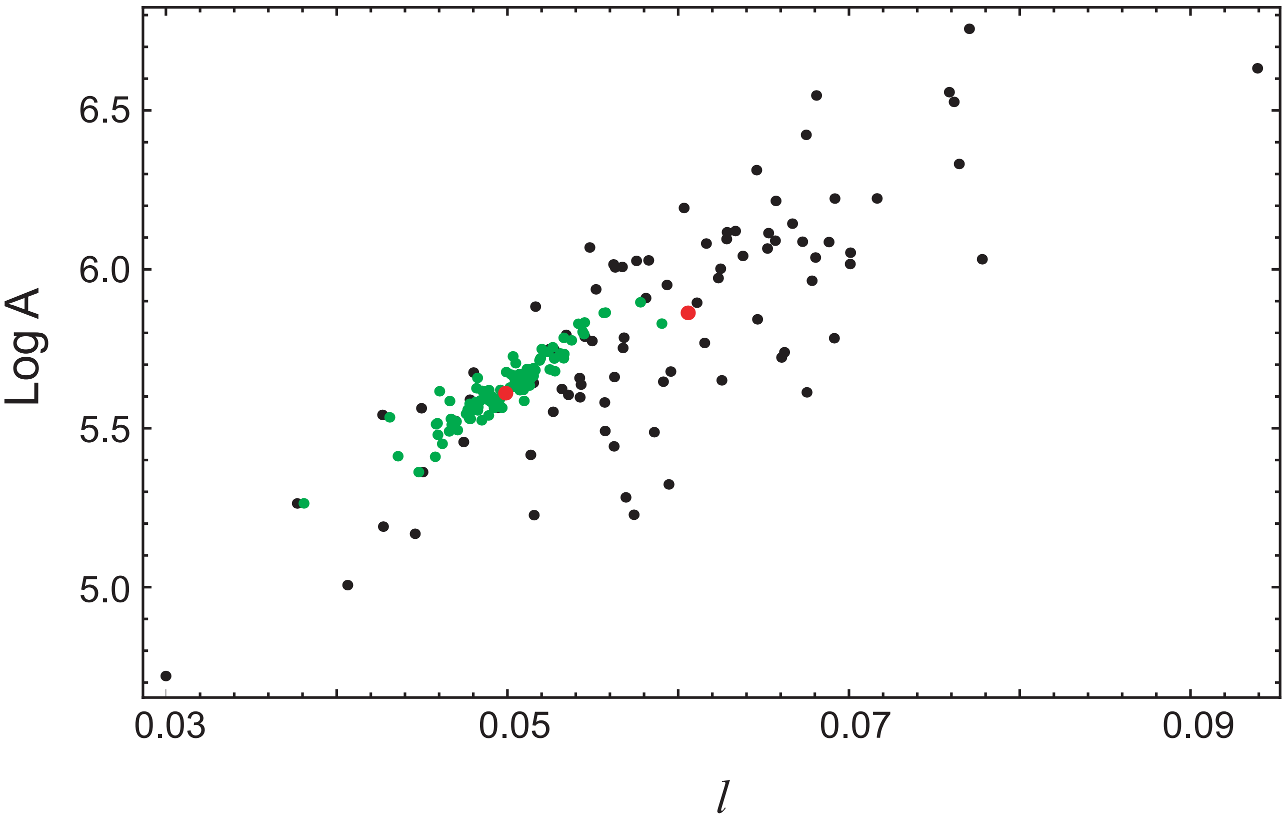

Figure S1. Parameters A and l of exponential approximation of individual Bcd profiles for the basic (black dots) and the alternative (green dots) normalization methods. The parameters corresponding to the median Bcd profiles in the two sets are highlighted by red dots.
