# Supplementary material for: Mechanisms of gap gene expression canalization in the Drosophila blastoderm
Source: BMC Syst Biol. 2011 Jul 28;5:118. doi: 10.1186/1752-0509-5-118 (PMC3398401; doi:10.1186/1752-0509-5-118)
Supplement: Additional file 2 — The bifurcation diagram for the new parameter values. [file 1752-0509-5-118-S2.PDF]

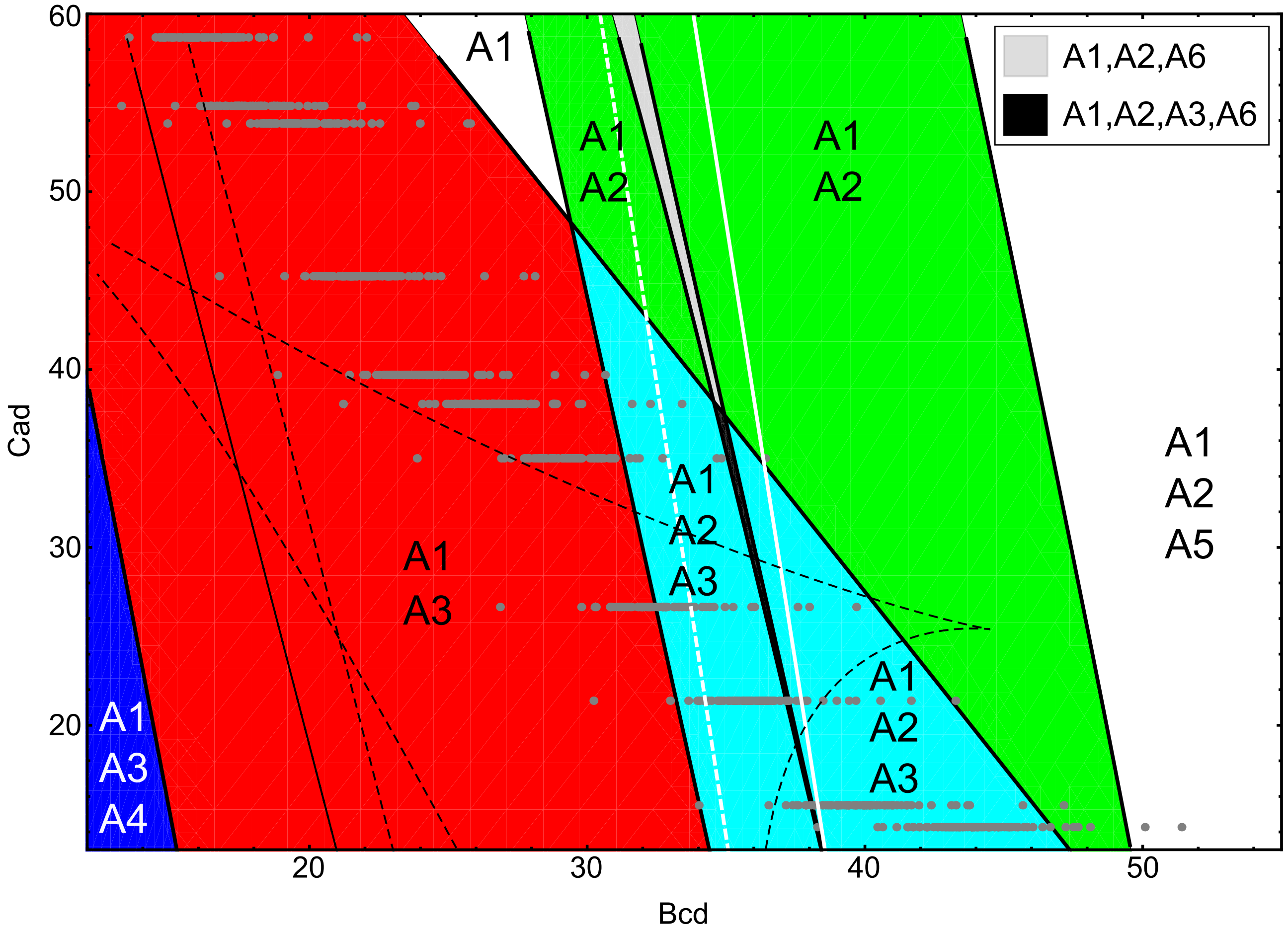

Figure S2. The bifurcation diagram on the Bcd–Cad plane for the simplified model from the main paper for the new parameter values (Additional file 10: Table S2). The figure shows the existence domains for point attractors A_1–A_6, described in the main paper. The boundaries (black solid lines) delimiting these domains represent positions of all bifurcations affecting the attractors. The black dashed lines show the positions of bifurcations affecting only saddle equilibria. The white dashed and solid lines are loci of points where the Gt concentration at attractor A_1 equals 50 and 150, respectively. Therefore, $A_1 = A_1^-$ to the left of the white dashed line, $A_1 = A_1^+$ to the right of the white solid line, and $A_1 = A_1^x$ between the lines. The gray dots have the same meaning as in Fig. 1B of the main paper but correspond to the alternatively normalized Bcd profiles.
