# Supplementary material for: Mechanisms of gap gene expression canalization in the Drosophila blastoderm
Source: BMC Syst Biol. 2011 Jul 28;5:118. doi: 10.1186/1752-0509-5-118 (PMC3398401; doi:10.1186/1752-0509-5-118)
Supplement: Additional file 3 — The spatial dependence of attractors in the shorted model for the median Bcd profile. [file 1752-0509-5-118-S3.PDF]

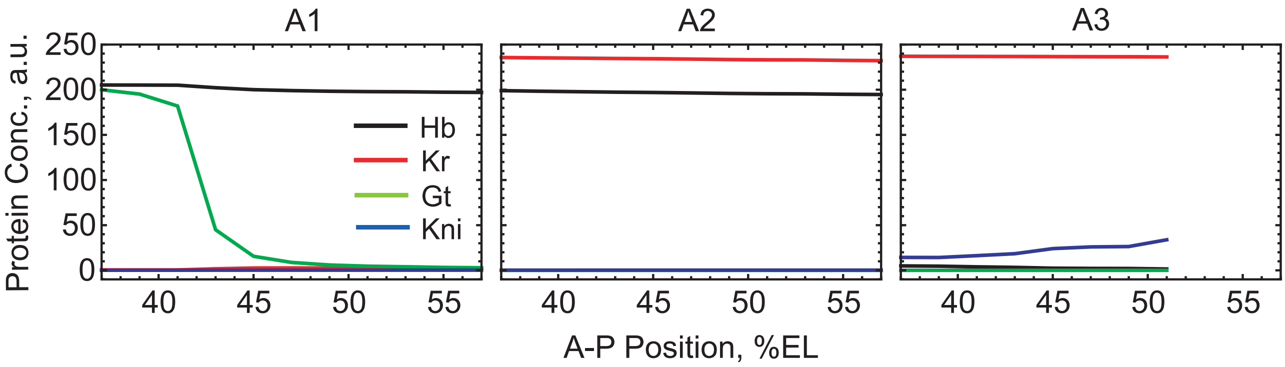

Figure S3. Spatial dependence of attractors A_1–A_3 in the simplified model for the median Bcd profile. The curves are linearly interpolated from calculations at eleven nuclei positions from 37%EL to 57%EL with the 2% interval. Attractor A_4 has empty attraction basin in Ω for this Bcd profile.
