# Supplementary material for: Mechanisms of gap gene expression canalization in the Drosophila blastoderm
Source: BMC Syst Biol. 2011 Jul 28;5:118. doi: 10.1186/1752-0509-5-118 (PMC3398401; doi:10.1186/1752-0509-5-118)
Supplement: Additional file 4 — The spatial dependence of attractors in the shorted model with the new parameter values and for the median Bcd profile. [file 1752-0509-5-118-S4.PDF]

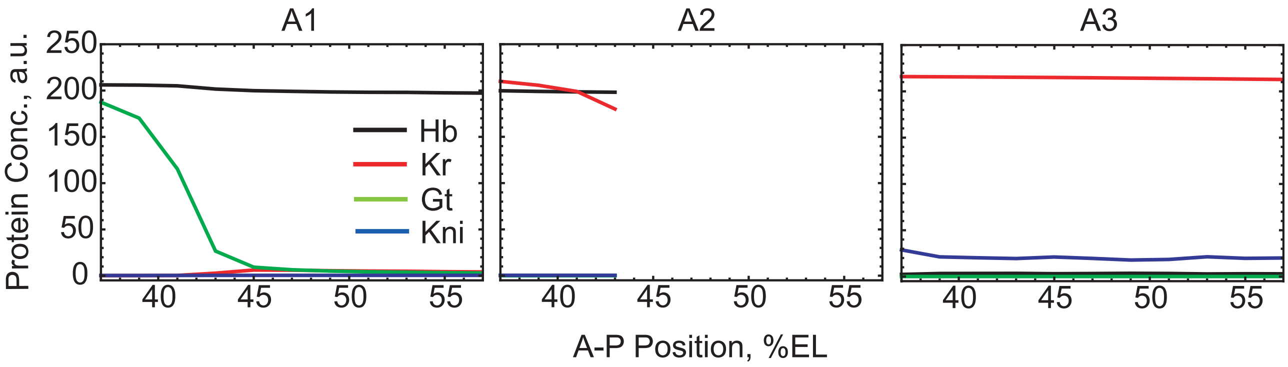

Figure S4. The spatial dependence of attractors A_1–A_3 in the model with the new parameter values (Additional file 10: Table S2) and for the median Bcd profile from the ensemble of the alternatively normalized Bcd profiles. The curves are linearly interpolated from calculations at eleven nuclei positions from 37%EL to 57%EL with the 2% interval. Attractors A_4–A_6 have empty attraction basins in Ω for this Bcd profile.
