# Supplementary material for: Mechanisms of gap gene expression canalization in the Drosophila blastoderm
Source: BMC Syst Biol. 2011 Jul 28;5:118. doi: 10.1186/1752-0509-5-118 (PMC3398401; doi:10.1186/1752-0509-5-118)
Supplement: Additional file 5 — Detailed description of the bifurcations in the model (the text contains reference to Figure S5). [file 1752-0509-5-118-S5.PDF]

Protocol S1: Bifurcations in the simplified model

We describe here in more details bifurcations appearing in the simplified model (2) from the main text in a constraint part of the Bcd–Cad plane, which is the plane with coordinates v^{Bcd} and v^{Cad}. The goal of bifurcation analysis is to have a global understanding of the behavior of a system with respect to the parameters. In our case, we explore how the equilibria in the model behave when we change concentrations of Bcd and Cad. As the result, we divide the Bcd–Cad plane into regions in which the number and type of equilibria remain constant. One can say that the qualitative behavior of the system does not change inside each such region. The analysis was done with the help of the AUTO software [1].

We use notation S for saddle equilibria and A for attractors found in the model. The stability of an equilibrium is determined by the eigenvalues of the Jacobian calculated at the equilibrium. Saddle is an equilibrium point whose Jacobian has eigenvalues with at least one positive and one negative real parts. Attractor is an equilibrium point whose Jacobian has all eigenvalues with negative real parts, i.e. it attracts solution trajectories locally along four directions in the phase space locally specified by the eigenvectors in the model. To track down how many eigenvalues have positive and how many have negative real parts, we use notation (i, j), where i is the number of eigenvalues with positive real parts and j is the number of eigenvalues with negative real parts ($i+j = 4$ is the total number of eigenvalues in the system). Thus, saddle $S(i, j)$ is an equilibrium point that attracts solution trajectories along j directions and repels along i directions in the phase space, as specified by corresponding eigenvectors. In these notations, attractors are always written as $A(0, 4)$.

A nondegenerate equilibrium is an equilibrium point whose Jacobian has all eigenvalues with nonzero real parts. We found nondegenerate equilibria of three types in the model: attractor $A(0, 4)$, and saddles $S(1, 3)$ and $S(2, 2)$.

Bifurcations occur when the stability of an equilibrium changes as we continue the equilibrium point by varying parameter $(v^{\mathrm{Bcd}}, v^{\mathrm{Cad}})$. At the bifurcation point, one or more of the eigenvalues have zero real part. We used the following algorithm to calculate the locations of all bifurcations on the Bcd–Cad plane. We started from an arbitrary value of $(v^{\mathrm{Bcd}}, v^{\mathrm{Cad}})$ and found an equilibrium point in the model by using the Newton–Raphson algorithm. Next, we continued this equilibrium point by varying either v^{Bcd} or v^{Cad} until we found a saddle–node or a Hopf bifurcation at some location on the Bcd–Cad plane. Then, we continued this location obtaining a curve on the Bcd–Cad plane such that all points on that curve exhibited the same bifurcation. In this way, we found all possible types of bifurcations, thus deriving the parametric portrait (bifurcation diagram) for the model (Additional file 6: Figure S5). The portrait is a subdivision of the Bcd–Cad plane into regions in which no bifurcation occurs.

As seen in the figure, there are in total four different types of bifurcations in the constrained part of the Bcd–Cad plane:

- saddle–node/saddle–saddle (fold) bifurcation,
- Hopf bifurcation,

- cusp bifurcation, and
- Bogdanov–Takens bifurcation.

Saddle–node/saddle–saddle (fold) bifurcation

This bifurcation happens when exactly one eigenvalue of the Jacobian at the equilibrium point has zero real part. Two equilibria (attractor and saddle or two saddles) either come into existence simultaneously or annihilate each other when the bifurcation curve is crossed on the Bcd–Cad plane depending on the direction of the crossing.

There are two types of fold bifurcation in the model (Additional file 6: Figure S5). Saddles $S(1,3)$ and $S(2,2)$ annihilate each other going from region 7 to 6, 14 to 3, 16 to 15, 7 to 8, 12 to 11, 10 to 18, 3 to 15, 14 to 16, 11 to 16, 2 to 3, 13 to 3, 12 to 14, and 17 to 16. A saddle $S(1,3)$ and an attractor $A(0,4)$ annihilate each other going from region 8 to 9, 11 to 10, 16 to 18, 6 to 5, 3 to 4, 5 to 4, and 6 to 3.

Hopf bifurcation

If we cross a Hopf bifurcation curve (one of the two blue curves in Additional file 6: Figure S5), one of the equilibrium points loses/gains stability along two directions in the phase space specified by corresponding eigenvectors, as a pair of complex conjugate eigenvalues of the Jacobian at the equilibrium crosses the imaginary axis of the complex plane. Since we have four eigenvalues for each equilibrium, one can have the following two changes at the Hopf bifurcation: $A(0,4)$ becoming $S(2,2)$, or $S(1,3)$ becoming $S(3,1)$ (or vice versa). We have only found $A(0,4)$, $S(2,2)$, and $S(1,3)$ in the model for the given range of Bcd and Cad concentrations, hence the $A(0,4)$-to-$S(2,2)$ change is the only possible outcome of Hopf bifurcation in our study. This change happens going from region 9 to 10, 8 to 11, 7 to 12, 6 to 13, and 1 to 2 (Additional file 6: Figure S5). Under certain assumptions, a limit cycle forms from the equilibrium point at the Hopf bifurcation.

Cusp bifurcation

At the point where cusp bifurcation occurs (red arrows in Additional file 6: Figure S5), three eigenvalues merge into one eigenvalue, and two branches of saddle–node bifurcation curve meet tangentially. In our case, two saddles of $S(2,2)$ type and one $S(1,3)$ come together leaving just one $S(2,2)$ after the merging at the bifurcation point. It happens in Additional file 6: Figure S5 going through the cusp bifurcation points from region 17 to 16 and from region 2 to 3.

Bogdanov–Takens

At the point where Bogdanov–Takens bifurcation occurs (black arrows in Additional file 6: Figure S5), two eigenvalues of the Jacobian at an equilibrium point have real part equal to

zero. What follows is a short description of this bifurcation in terms of equilibria only (for a complete description of Bogdanov–Takens bifurcation refer to [2, Chapter 8]). The Bogdanov–Takens bifurcation point is the meeting place of saddle–node bifurcation curve (black in the figure) and Hopf bifurcation curve (blue in the figure). It occurs between regions 6, 3, and 13 and between regions 1, 2, and 3. Going from region 6 to 13, $A(0,4)$ changes to $S(2,2)$ and then, going from 13 to 3, that $S(2,2)$ is annihilated with $S(1,3)$. Therefore, going from region 6 to region 3 through the Bogdanov–Takens bifurcation point, $A(0,4)$ is simultaneously changed to $S(2,2)$ and annihilated with $S(1,3)$.

References

[1] AUTO: Software for continuation and bifurcation problems in ordinary differential equations (http://indy.cs.concordia.ca/auto)

[2] Yu. A. Kuznetsov. Elements of Applied Bifurcation Theory (2nd ed.), Springer–Verlag, NY–Berlin–Heidelberg, 1998
