# Supplementary material for: Mechanisms of gap gene expression canalization in the Drosophila blastoderm
Source: BMC Syst Biol. 2011 Jul 28;5:118. doi: 10.1186/1752-0509-5-118 (PMC3398401; doi:10.1186/1752-0509-5-118)
Supplement: Additional file 6 — The bifurcation diagram with more details. [file 1752-0509-5-118-S6.PDF]

Region	#A	#S(1,3)	#S(2,2)	Region	#A	#S(1,3)	#S(2,2)	Region	#A	#S(1,3)	#S(2,2)
1	4	4	1	7	4	5	2	13	3	4	2
2	3	4	2	8	4	4	1	14	3	4	2
3	3	3	1	9	3	3	1	15	3	2	0
4	2	2	1	10	2	3	2	16	3	3	1
5	3	3	1	11	3	4	2	17	3	4	2
6	4	4	1	12	3	5	3	18	2	2	1

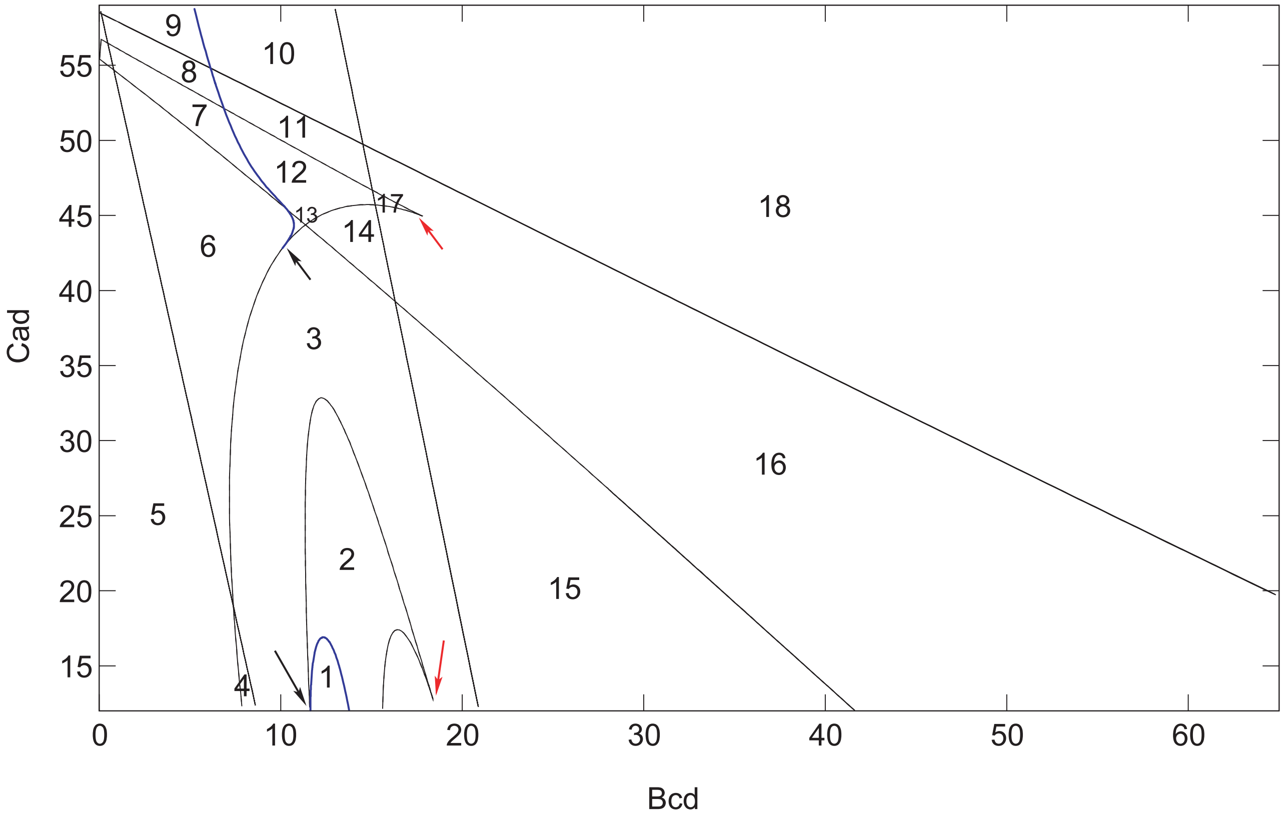

Figure S5. The bifurcation diagram from Fig. 3 of the main paper with more details. The black curves correspond to the saddle–node or saddle–saddle bifurcations, and the blue ones to Hopf bifurcations. The red arrows mark the locations of cusp bifurcation, and the black ones show the Bogdanov–Takens bifurcations. All regions separated by the bifurcation curves are numbered. For each numbered region, the tables over the plot present the total numbers of point attractors, saddles $S(1,3)$, and saddles $S(2,2)$. More details are given in Additional file 5: Protocol S1.
