# Supplementary material for: Mechanisms of gap gene expression canalization in the Drosophila blastoderm
Source: BMC Syst Biol. 2011 Jul 28;5:118. doi: 10.1186/1752-0509-5-118 (PMC3398401; doi:10.1186/1752-0509-5-118)
Supplement: Additional file 7 — The existence domains on the Bcd-Cad plane for attractors A1-A4 following from calculations at discrete spatial positions. [file 1752-0509-5-118-S7.PDF]

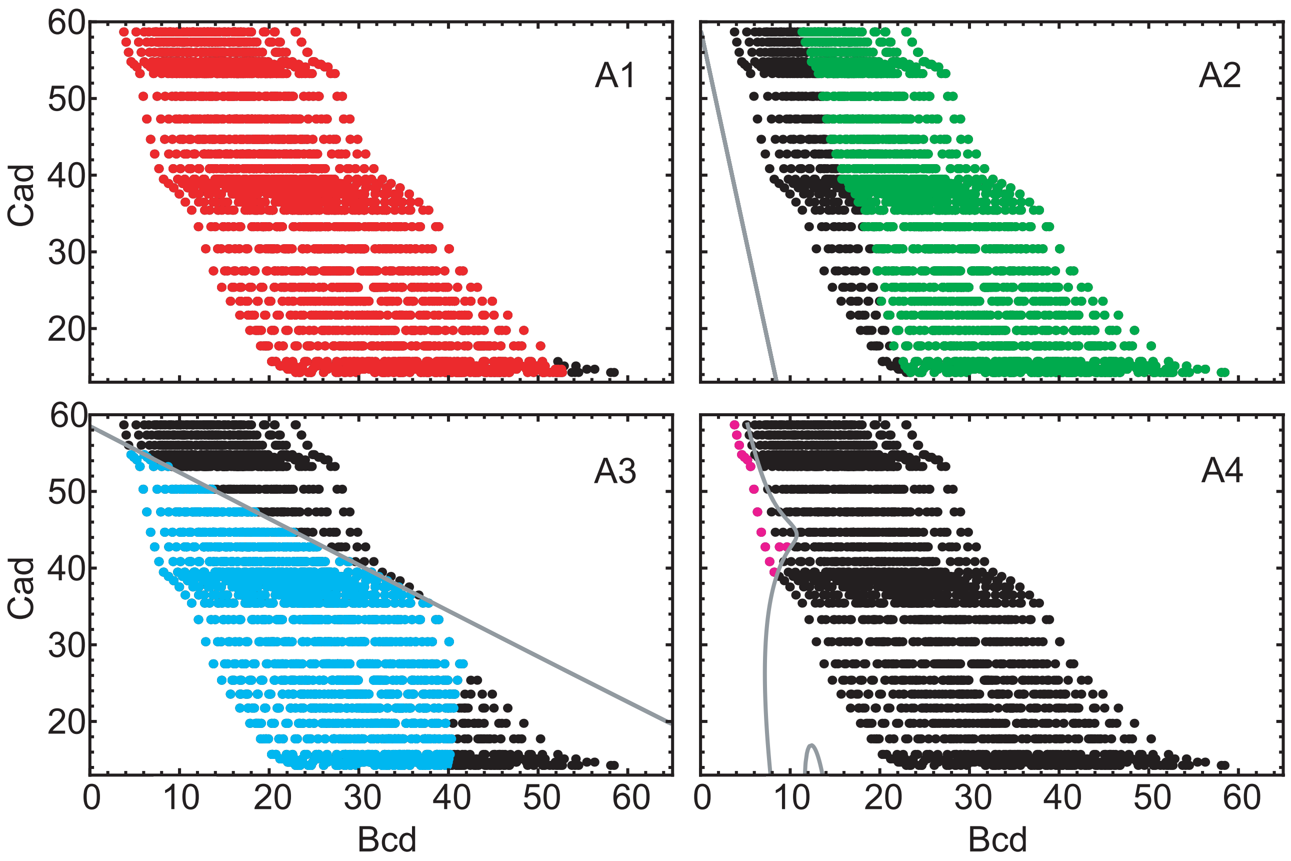

Figure S6. Existence domains on the Bcd–Cad plane for attractors A_1–A_4 following from calculations at discrete spatial positions. The dots in each panel have the same meaning as in Fig. 1B of the main paper, except that they are obtained not from 11 but from 30 positions equally spaced in the range 37–57%EL. For the $(v^{\mathrm{Bcd}}, v^{\mathrm{Cad}})$ parameter value at each dot, the attraction basins for A_1–A_4 were calculated in Ω. The dots in each panel are black if the attraction basin for the corresponding attractor is empty in Ω and color otherwise. The gray lines are the boundaries of existence domains for corresponding attractors predicted by the bifurcation analysis (see Fig. 3 in the main paper). The boundaries between the color-dot and the black-dot regions which are well approximated by the gray lines correspond to situations when the attractors stop to exist due to bifurcations. The boundaries which stay apart of the gray lines correspond to situations when the attractors have a nonempty attraction basin in the phase space, but this basin is disjoint with Ω.
