# Supplementary material for: Mechanisms of gap gene expression canalization in the Drosophila blastoderm
Source: BMC Syst Biol. 2011 Jul 28;5:118. doi: 10.1186/1752-0509-5-118 (PMC3398401; doi:10.1186/1752-0509-5-118)
Supplement: Additional file 8 — The distribution of Bcd profiles over the four mechanisms of hb border formation and over solution classes I-III. [file 1752-0509-5-118-S8.PDF]

Table S1. The distribution of 88 Bcd profiles in the ensemble over four mechanisms of *hb* border formation, described in Fig. 4 of the main paper, and over solution classes I–III, described in Fig. 2 of the main paper. The mechanisms are labeled as follows: attractor–attractor switch (AA; Fig. 4A), attractor–manifold switch (AM; Fig. 4B), manifold–attractor switch (MA; Fig. 4C), and manifold–manifold switch (MM; Fig. 4D).

	AA	AM	MA	MM
class I	37	2	0	0
class II	9	0	1	0
class III	20	18	0	1
