# Supplementary material for: Mechanisms of gap gene expression canalization in the Drosophila blastoderm
Source: BMC Syst Biol. 2011 Jul 28;5:118. doi: 10.1186/1752-0509-5-118 (PMC3398401; doi:10.1186/1752-0509-5-118)
Supplement: Additional file 9 — The response curve for the normalized individual Bcd profiles instead of their exponential approximations. [file 1752-0509-5-118-S9.PDF]

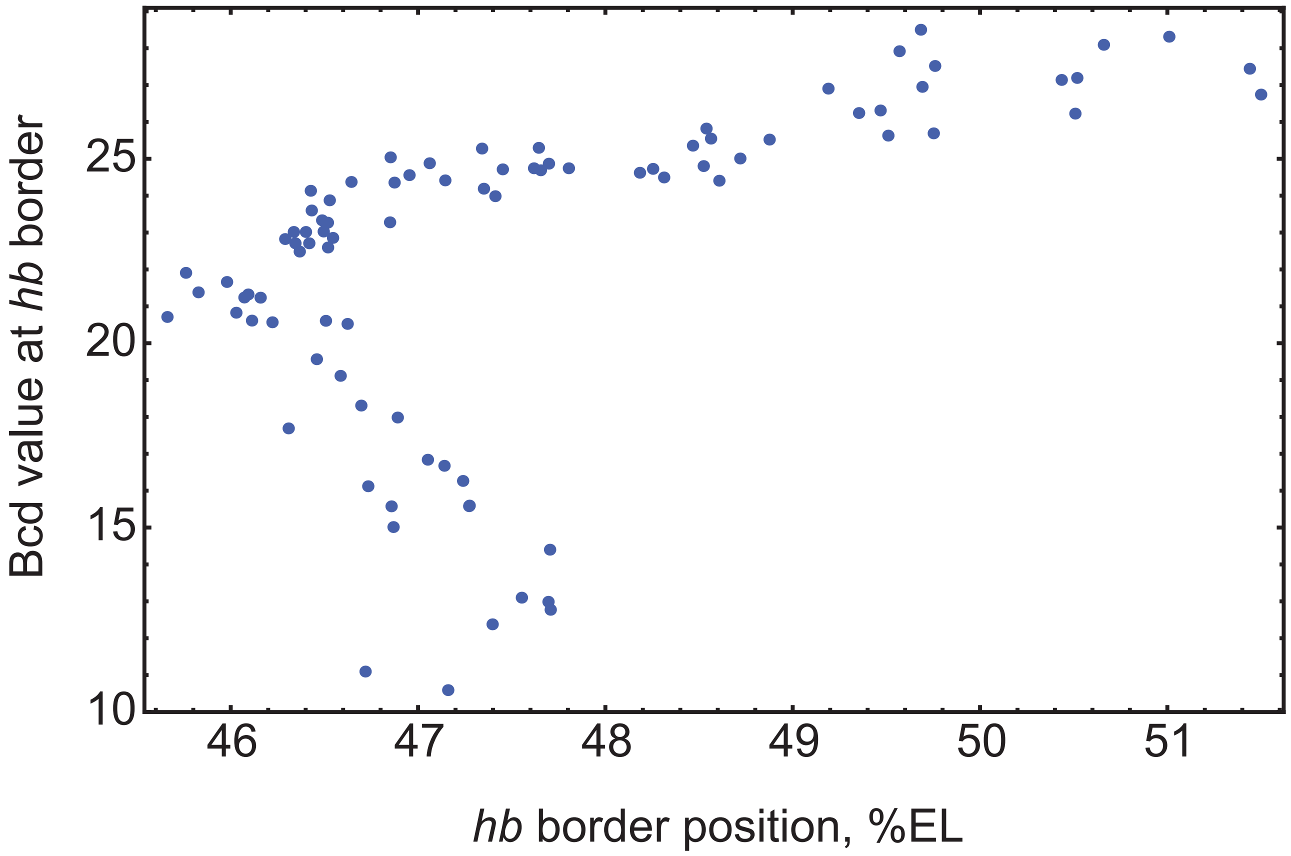

Figure S7. The response curve (analog of Fig. 6B from the main paper) for the normalized individual Bcd profiles instead of their exponential approximations.
