# Supplementary material for: Mechanisms of gap gene expression canalization in the Drosophila blastoderm
Source: BMC Syst Biol. 2011 Jul 28;5:118. doi: 10.1186/1752-0509-5-118 (PMC3398401; doi:10.1186/1752-0509-5-118)
Supplement: Additional file 10 — New parameter values in the model obtained by optimization with a median Bcd profile from the Bcd data normalized by the alternative method. [file 1752-0509-5-118-S10.PDF]

Table S2. Parameter values obtained by optimization in the full model from the main paper with v_i^{Bcd} fixed to the median profile from the ensemble of individual Bcd profiles normalized by the alternative method. Values $h^a = -2.5$ were fixed for all genes.

	T^{ab}									
	hb	Kr	gt	kni	m^a	E^a	F^a	R^a	D^a	λ^a
hb	0.019	−0.001	0.018	−0.118	0.028	0.006	0.002	15.00	0.164	0.072
Kr	−0.021	0.015	−0.095	−0.024	0.132	0.019	−0.121	15.00	0.200	0.069
gt	−0.027	−0.177	0.003	−0.002	0.207	0.021	−0.010	15.00	0.079	0.075
kni	−0.056	−0.004	−0.016	0.014	0.035	0.017	−0.156	15.00	0.200	0.058
