# Supplementary material for: Mechanisms of gap gene expression canalization in the Drosophila blastoderm
Source: BMC Syst Biol. 2011 Jul 28;5:118. doi: 10.1186/1752-0509-5-118 (PMC3398401; doi:10.1186/1752-0509-5-118)
Supplement: Additional file 11 — The solutions of the full model equations and their simplified version for the new parameter values. [file 1752-0509-5-118-S11.PDF]

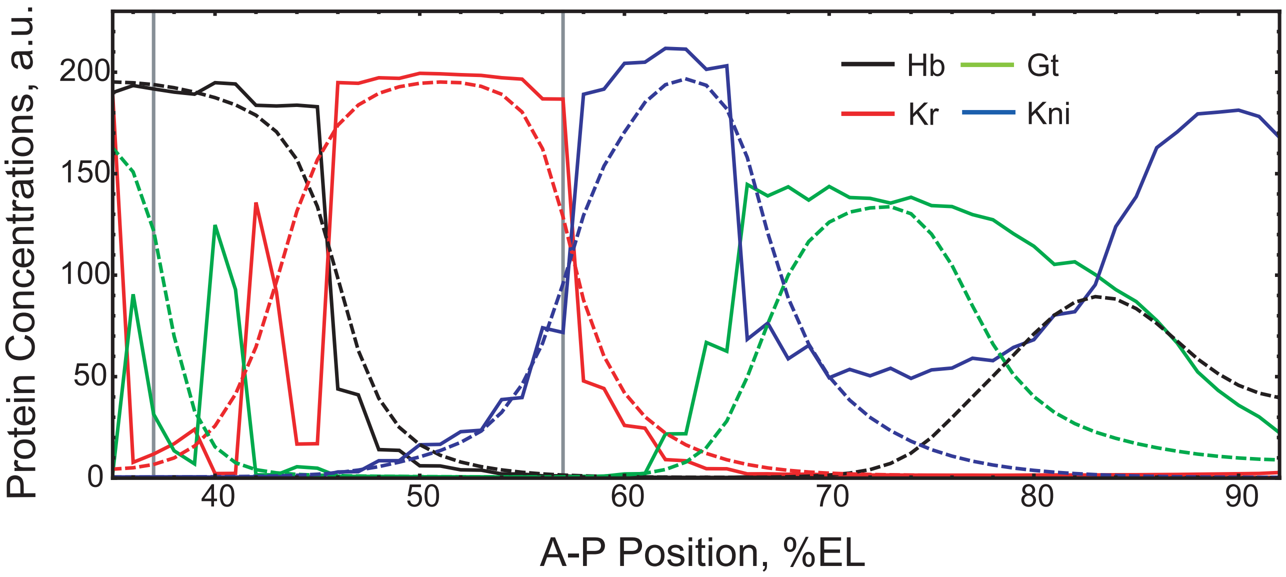

Figure S8. The solutions of the full model equations from the main text (dashed lines) and their simplified version (solid lines) for new parameter values (Additional file 10: Table S2) at time class 6, for the biological initial conditions and the median Bcd profile from the Bcd ensemble normalized by the alternative method. The vertical gray lines demarcate the spatial domain 37–57%EL chosen for the dynamical analysis of *hb* border formation.
