# Supplementary material for: Mechanisms of gap gene expression canalization in the Drosophila blastoderm
Source: BMC Syst Biol. 2011 Jul 28;5:118. doi: 10.1186/1752-0509-5-118 (PMC3398401; doi:10.1186/1752-0509-5-118)
Supplement: Additional file 12 — The bifurcation diagram for the new parameter values with more details. [file 1752-0509-5-118-S12.PDF]

Region	#A	#S(1,3)	#S(2,2)	Region	#A	#S(1,3)	#S(2,2)	Region	#A	#S(1,3)	#S(2,2)
1	2	2	1	9	2	3	2	17	2	2	1
2	2	3	2	10	3	3	1	18	2	3	2
3	2	2	1	11	3	4	2	19	2	2	1
4	1	1	1	12	3	3	1	20	3	2	0
5	2	2	1	13	3	3	1	21	2	3	2
6	3	3	1	14	3	4	2	22	2	4	3
7	2	2	1	15	3	3	1	23	4	4	1
8	3	3	1	16	2	3	2	24	4	5	2
								25	4	4	1

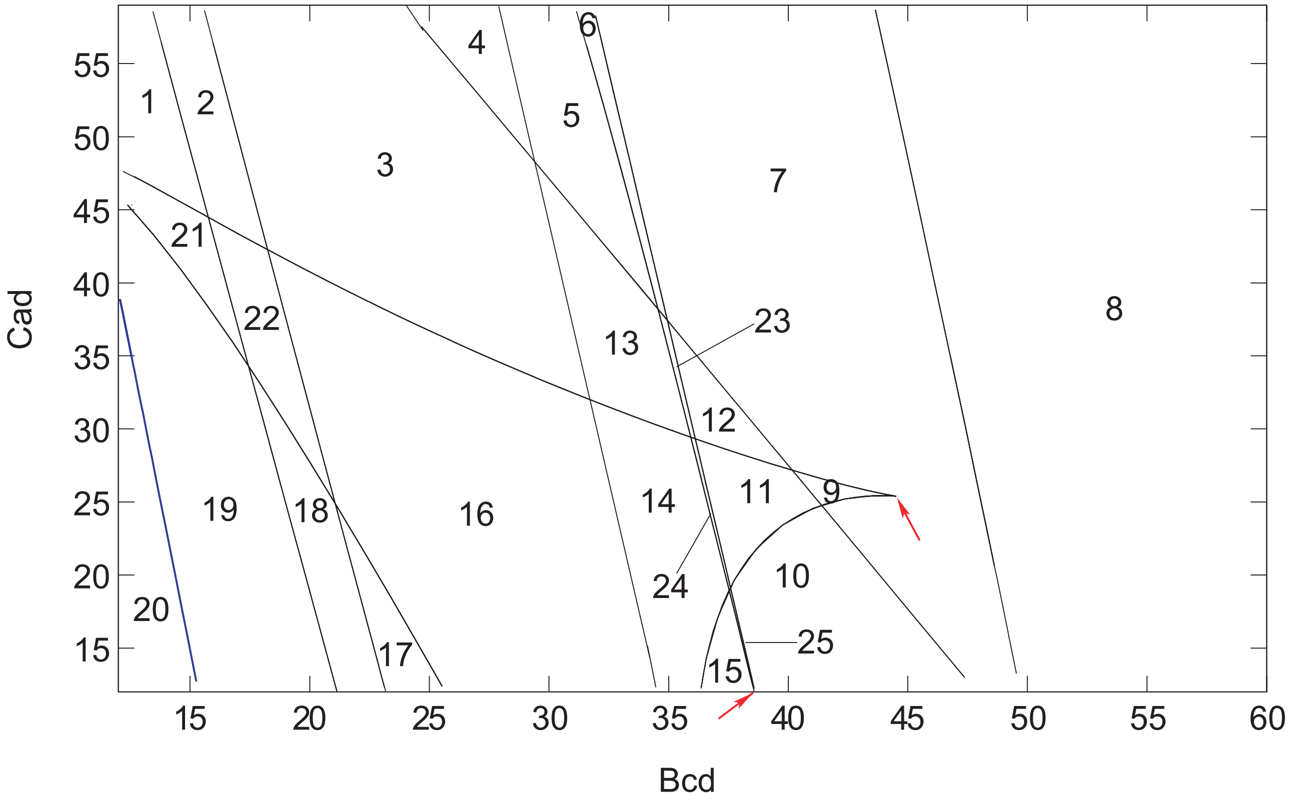

Figure S9. The bifurcation diagram from Additional file 2: Figure S2 with more details. The black curves correspond to the saddle–node or saddle–saddle bifurcations, and the blue one to Hopf bifurcation. The red arrows mark the locations of cusp bifurcation. All regions separated by the bifurcation curves are numbered. For each numbered region, the tables over the plot present the total numbers of point attractors, saddles $S(1,3)$, and saddles $S(2,2)$.
