# Supplementary material for: Mechanisms of gap gene expression canalization in the Drosophila blastoderm
Source: BMC Syst Biol. 2011 Jul 28;5:118. doi: 10.1186/1752-0509-5-118 (PMC3398401; doi:10.1186/1752-0509-5-118)
Supplement: Additional file 13 — Detailed description of the bifurcations for the new parameter values (the text contains reference to Figure S9). [file 1752-0509-5-118-S13.PDF]

Protocol S2: Bifurcations in the simplified model with the new parameter values

We provide here more details on bifurcations appearing in the simplified model (2) from the main text with the new parameter values (Additional file 10: Table S2), in a constraint part of the Bcd–Cad plane. The notations and short definitions of bifurcation types are given in Additional file 5: Protocol S1.

The parametric portrait is shown in Additional file 12: Figure S9 of Supplementary Material. We found three different types of bifurcations:

- saddle–node/saddle–saddle (fold) bifurcation (black lines in the figure),
- Hopf bifurcation (blue line), and
- cusp bifurcation (points marked by red arrows).

We can see in the figure two different types of fold bifurcations: (1) $S(1,3)$ and $S(2,2)$ annihilate each other going from region 2 to 1, 2 to 3, 9 to 7, 22 to 21, 21 to 19, 11 to 12, 11 to 10, 11 to 6, 14 to 6, 21 to 1, 22 to 2, 14 to 13, 14 to 15, 24 to 23, 24 to 25, 18 to 19, 22 to 18, 18 to 17, 16 to 17, 22 to 16, and 16 to 3, and (2) $S(1,3)$ and $A(0,4)$ annihilate each other going from region 3 to 4, 5 to 4, 6 to 5, 6 to 7, 8 to 7, 10 to 7, 11 to 9, 13 to 5, 13 to 3, 13 to 7, 23 to 13, 23 to 12, 24 to 14, 24 to 11, 25 to 15, 25 to 10, 6 to 23, and 14 to 16.

The Hopf bifurcation is associated with the change of an attractor to a saddle of type $S(2,2)$. Namely, $A(0,4)$ becomes $S(2,2)$ going from region 20 to 19.
