# Supplementary material for: Mechanisms of gap gene expression canalization in the Drosophila blastoderm
Source: BMC Syst Biol. 2011 Jul 28;5:118. doi: 10.1186/1752-0509-5-118 (PMC3398401; doi:10.1186/1752-0509-5-118)
Supplement: Additional file 14 — The existence domains on the Bcd-Cad plane for attractors A1-A6 in the model with the new parameter values following from calculations at eleven spatial positions. [file 1752-0509-5-118-S14.PDF]

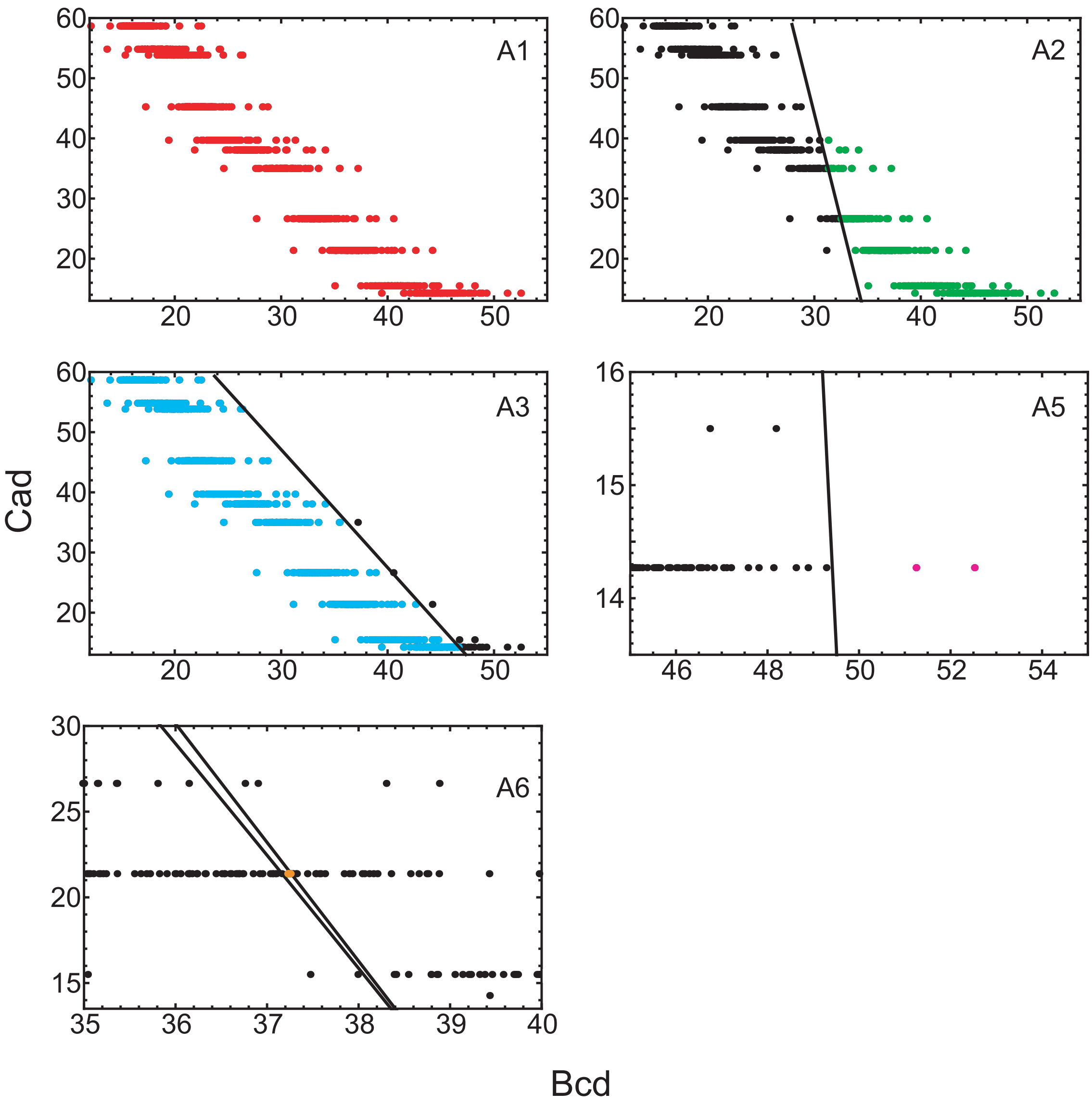

Figure S10. The existence domains on the Bcd–Cad plane for attractors A_1–A_6 in the model with the new parameter values (Additional file 10: Table S2) following from calculations at eleven spatial positions. The dots in each panel have the same meaning as in Fig. 1B of the main paper, except that they correspond to the alternatively normalized Bcd profiles. The dots in each panel are black if the attraction basin for the corresponding attractor is empty in Ω and color otherwise. The black lines are boundaries of the existence domains for corresponding attractors predicted by the bifurcation analysis (see Additional file 12: Figure S9). The attraction basin for attractor A_4 has not been found in the calculations. Attractors A_5 and A_6 have nonempty attraction basins only for a few spatial positions and Bcd profiles, so we zoomed the informative regions of the plane in corresponding panels.
