# Supplementary material for: Mechanisms of gap gene expression canalization in the Drosophila blastoderm
Source: BMC Syst Biol. 2011 Jul 28;5:118. doi: 10.1186/1752-0509-5-118 (PMC3398401; doi:10.1186/1752-0509-5-118)
Supplement: Additional file 15 — Classification results for the Bcd profiles in the case of the alternative normalization method. [file 1752-0509-5-118-S15.PDF]

Table S3. Classification results in the case of the alternative normalization method. The table shows the distribution of 89 Bcd profiles over the three mechanisms of *hb* border formation (columns), over the solution classes in the model with these Bcd profiles (upper three rows), and over the type of switch between the attraction basins of attractors A_1–A_3 that happens across the nuclei surrounding the *hb* border position (lower three rows). The mechanisms are labeled as in Additional file 8: Table S1.

	AA	MA	MM
class I	19	1	0
class II	24	5	4
class III	2	29	5
$A^1 \to A^3$	43	35	7
$A^2 \to A^3$	2	0	0
$A^1 \to A^1$	0	0	2
