# Supplementary material for: Mechanisms of gap gene expression canalization in the Drosophila blastoderm
Source: BMC Syst Biol. 2011 Jul 28;5:118. doi: 10.1186/1752-0509-5-118 (PMC3398401; doi:10.1186/1752-0509-5-118)
Supplement: Additional file 16 — The spatial configuration of attraction basins in the model with the new parameter values. [file 1752-0509-5-118-S16.PDF]

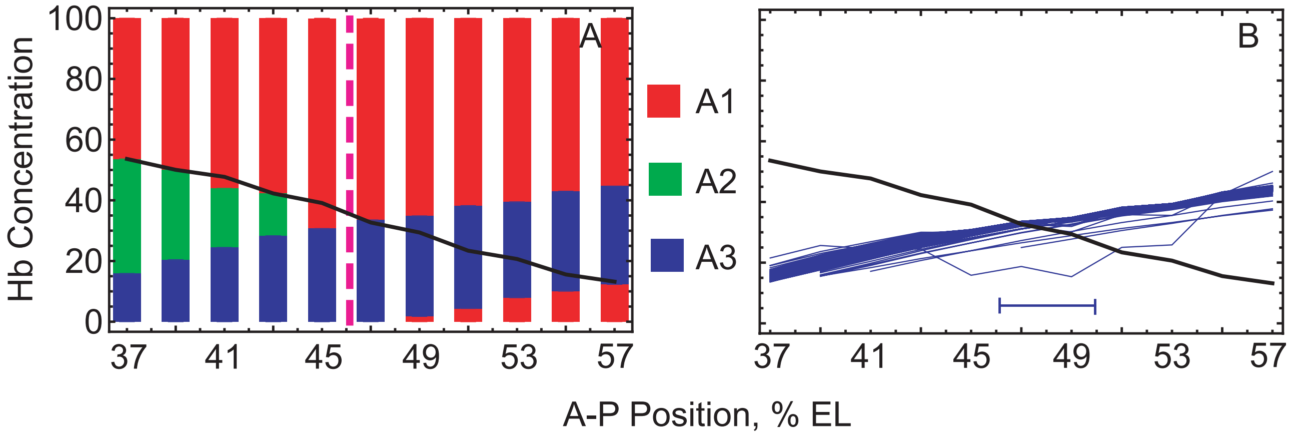

Figure S11. Analog of Fig. 5A,C from the main text but for the new parameter values (Additional file 10: Table S2) and for the Bcd ensemble normalized by the alternative method. The spatial configuration of attraction basins in (A) is shown for the median Bcd profile. Panel (B) presents results for 87 Bcd profiles associated with the intersection of the initial Hb profile and the basin boundary for attractor A_3. The line segment at the bottom of (B) shows the range of *hb* border positions in the solutions of the simplified model equations corresponding to these Bcd profiles. Two Bcd profiles were excluded from this analysis because for these profiles there was no transition between different attraction basins across the nuclei surrounding the *hb* border position.
