# Supplementary material for: Mechanisms of gap gene expression canalization in the Drosophila blastoderm
Source: BMC Syst Biol. 2011 Jul 28;5:118. doi: 10.1186/1752-0509-5-118 (PMC3398401; doi:10.1186/1752-0509-5-118)
Supplement: Additional file 17 — The response curve for the new parameter values. [file 1752-0509-5-118-S17.PDF]

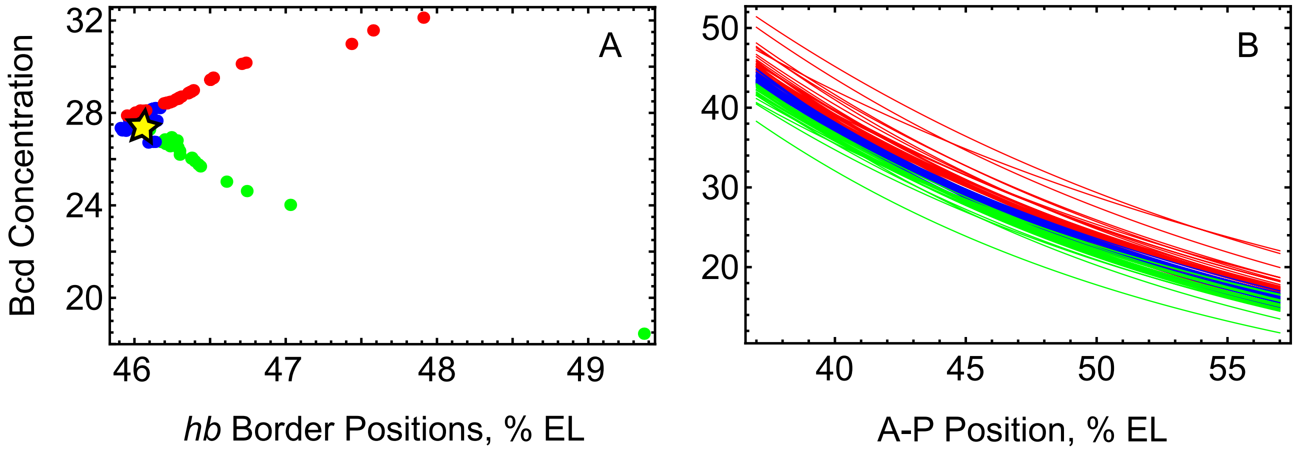

Figure S12. Analog of Fig. 6B from the main text but for the new parameter values (Additional file 10: Table S2) and for the Bcd ensemble normalized by the alternative method. The response curve in (A) is calculated for the full model (1) from the main text, and the Bcd profiles from the ensemble are shown in (B).
