# Supplementary material for: Mechanisms of gap gene expression canalization in the Drosophila blastoderm
Source: BMC Syst Biol. 2011 Jul 28;5:118. doi: 10.1186/1752-0509-5-118 (PMC3398401; doi:10.1186/1752-0509-5-118)
Supplement: Additional file 18 — Schematic illustration of the first canalization mechanism. [file 1752-0509-5-118-S18.PDF]

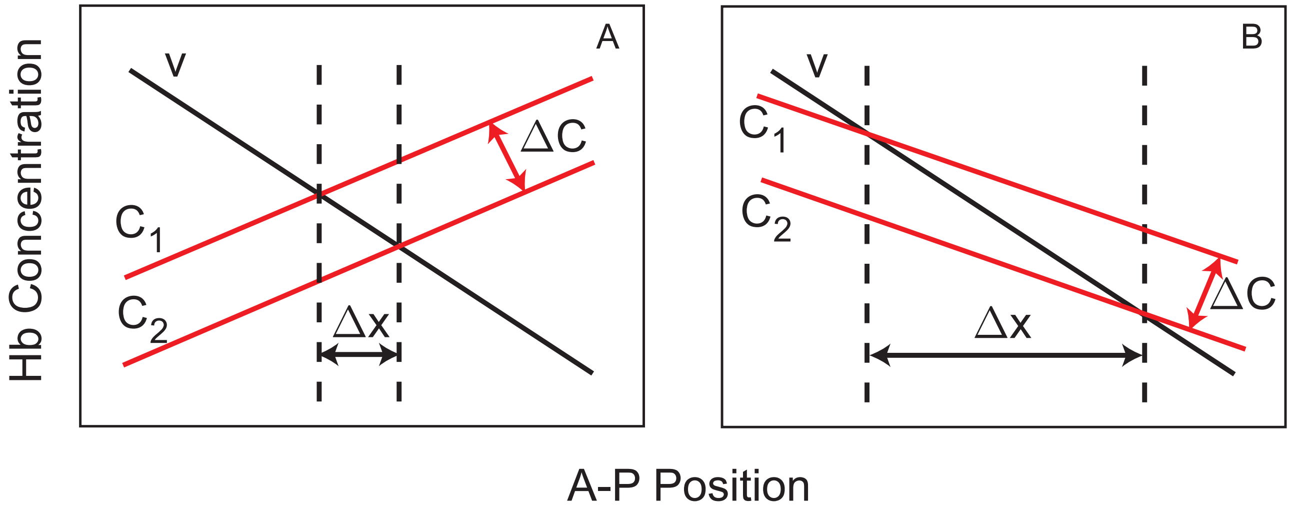

Figure S13. Schematic illustration of the first mechanism of canalization. The line v in the panels represents the initial Hb profile, while C_1 and C_2 are attraction basin boundaries for two different Bcd profiles. Δx is the spatial variance of the intersection points, which encode the actual *hb* border positions. Despite the fact that the absolute variance ΔC of basin boundaries in the protein concentration space is the same in both panels, the positional variances Δx are drastically different in the two situations.
