# Supplementary material for: Mechanisms of gap gene expression canalization in the Drosophila blastoderm
Source: BMC Syst Biol. 2011 Jul 28;5:118. doi: 10.1186/1752-0509-5-118 (PMC3398401; doi:10.1186/1752-0509-5-118)
Supplement: Additional file 19 — The regulatory analysis of the response curve (the Protocol contains Figures S14-S17). [file 1752-0509-5-118-S19.PDF]

Protocol S3: Regulatory analysis of response curve

We perform the regulatory analysis to identify genes in the gap gene network whose influence is responsible for the specific folding form of the *hb* response curve from Fig. 6B in the main text. We use the method for the analysis originally proposed in [4]. Below we describe in more details this method applied to the full model (1) from the main paper in our study.

In the vicinity of *hb* border, the Hb concentration profile in the solution sharply changes from a large concentration value to a small one as we go from anterior to posterior. This can only happen if the regulation function g in the equation for Hb takes values at the linearly decreasing part of its sigmoid graph in the vicinity of the border, because g is almost constant (either 1 or 0) away from this part for all values of its argument. Therefore, we can approximate the regulation function $g(u^{\mathrm{Hb}}(x,t)) \sim u^{\mathrm{Hb}}(x,t)$ in this vicinity, where $u^{\mathrm{Hb}}(x,t)$ describes the full regulatory input to *hb* expression in the model at A–P position x and time t:

$$u^{\mathrm{Hb}}(x,t) = \sum_{b=1}^{4} T^{hb\leftarrow b} v^{b}(x,t) + m^{\mathrm{Hb}} v^{\mathrm{Bcd}}(x) + E^{\mathrm{Hb}} v^{\mathrm{Cad}}(x,t) + F^{\mathrm{Hb}} v^{\mathrm{Tll}}(x,t) + h^{\mathrm{Hb}}.$$

The functions $v^{b}(x,t)$ were obtained by linearly interpolating in space the solution $v_i^{b}(t)$ of the model equations, i.e., the discrete index i was turned to the continuous spatial coordinate x. The same was done for functions $v^{\mathrm{Cad}}(x,t)$ and $v^{\mathrm{Tll}}(x,t)$, and $v^{\mathrm{Bcd}}(x)$ was described in Methods of the main text.

As the full regulatory input is simply a sum of inputs from all individual factors (four gap proteins, including Hb as a self-regulator, Bcd, Cad, Tll, and the constant h^{Hb}), we can analyze which portion the individual inputs take in this sum. We can exclude Cad, Tll, and constant h^{Hb} from the regulatory analysis of the response curve, because these factors do not change for varying Bcd profile and, hence, their joint input can be assumed constant along the response curve. As we are mostly interested in explaining the folding part of the curve, we can further exclude Bcd and Hb itself from the analysis. Bcd activates *hb*, and, therefore, the rising Bcd concentration can only lead to posterior shift of *hb* border (in the absence of other regulators [3]). The Hb self-activation also tends to shift the border posteriorly, because it provides a larger input for larger Hb concentration. Thus, only the rest of the gap genes can be responsible for the non-monotone form of *hb* response curve, and we have the following individual inputs from their proteins:

$$u^{\mathrm{Hb,Kr}}(x,t) = T^{hb\leftarrow \mathrm{Kr}} v^{\mathrm{Kr}}(x,t),\ u^{\mathrm{Hb,Gt}}(x,t) = T^{hb\leftarrow \mathrm{Gt}} v^{\mathrm{Gt}}(x,t),\ u^{\mathrm{Hb,Kni}}(x,t) = T^{hb\leftarrow \mathrm{Kni}} v^{\mathrm{Kni}}(x,t).$$

In order to compare the individual inputs and their combinations to the full regulatory input for various Bcd profiles, we fix time at time class T6 and spatial coordinate x at the center of the interval $44.6\%\mathrm{EL} < x < 49.5\%\mathrm{EL}$. For most Bcd profiles from the ensemble, the *hb* border in the solutions lies inside this interval and the interval covers the vicinity of *hb* border between the 90%- and 10%-expression levels for Hb (Fig. S14). We select 66 Bcd profiles satisfying this condition. The approximation $g(u^{\mathrm{Hb}}) \sim u^{\mathrm{Hb}}$ is valid inside the interval for these Bcd profiles, and the interval center is a good point for the regulatory analysis of the response curve.

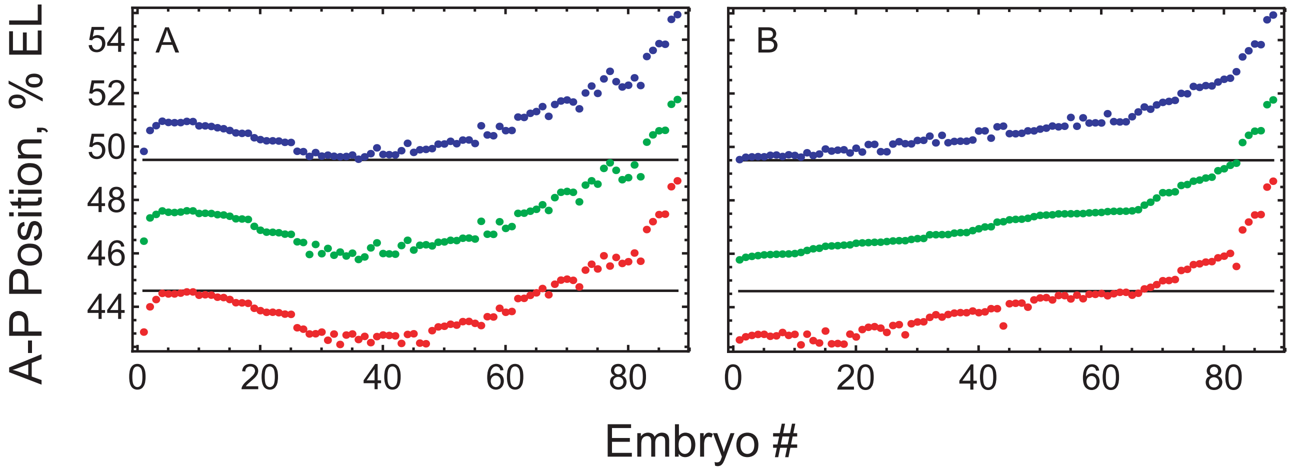

Figure S14. The choice of the spatial interval for regulatory analysis of the response curve. Each dot in the panels corresponds to the Hb component of solution in the full model (1) from the main text for each of the 88 Bcd profiles and shows the position where Hb in the solution is expressed at 10% (blue dots) or 90% (red dots) of its maximal concentration at the anterior end, and the *hb* border position in the solution (green dots). These positions are sorted in (A) according to the rising Bcd concentration at the *hb* border and in (B) according to the rising *hb* border position. The solid lines extract a spatial interval ($44.6\%\mathrm{EL} < x < 49.5\%\mathrm{EL}$) in which the Hb component of the solutions for most of the Bcd profiles is between 10%- and 90%-expression levels. 66 Bcd profiles satisfying this condition are chosen for the regulatory analysis.

The regulatory inputs from various factors to *hb* expression in the model are shown in Fig. S15 for the chosen Bcd profiles. The curves made of dots in this figure are in fact response curves in terms of regulatory inputs. The inputs from combinations of genes are calculated as a sum of the individual inputs from corresponding genes. We can see that the full input curve exhibits the folds. This Bcd dependence of the full input determines the folds of the *hb* response curve from Fig. 6B in the main text. As discussed above, the folds of the full input curve are determined by the influence from gap proteins Kr, Gt, and Kni, represented by the curve of grey dots in Fig. S15.

In order to further dissect main regulators responsible for the non-monotone form of this grey curve, we find curves in the figure which the grey one comes very close. We can see that, at small Bcd concentration values, it comes close to the red one (the lowermost arrow in the figure), which means that the influence of the three gap proteins is almost fully determined by the influence of proteins Gt and Kni for this Bcd concentration range. While these two curves stay close enough to each other, they also stay monotonously increasing for rising Bcd concentration. The grey curve exhibits a similar behavior for large Bcd concentration values (the uppermost arrow and above), where it almost coincide with the orange curve. This means

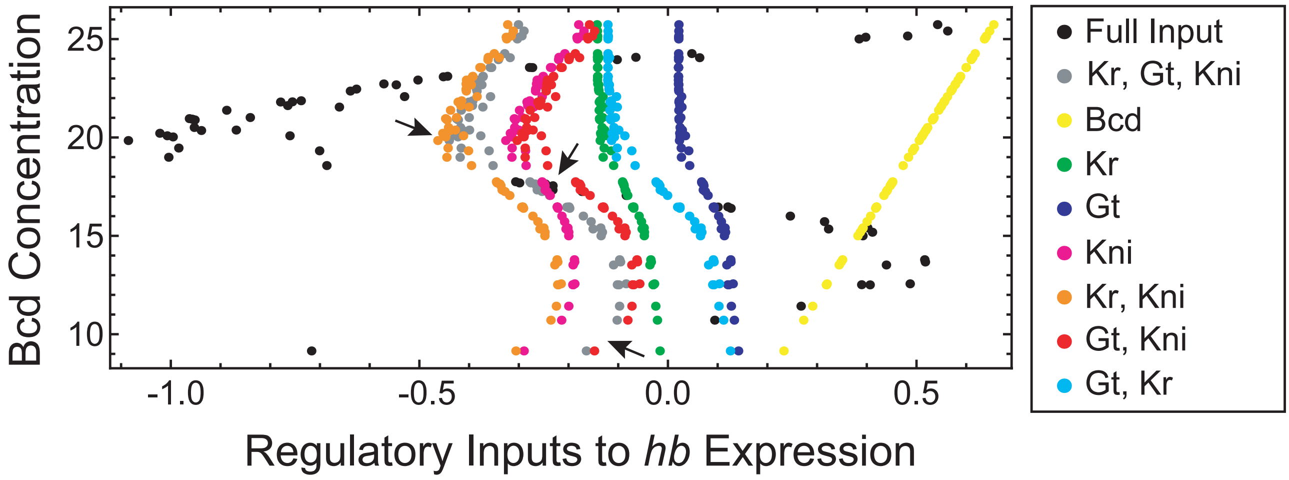

Figure S15. The response curve in terms of regulatory inputs to *hb* expression. The abscissa of each dot gives the regulatory input from a combination of genes (proteins) indicated in the plot legend to *hb* expression at the solution of full model equations (1) from the main text for a given Bcd profile and for fixed time and spatial position, and the ordinate gives the value of this Bcd concentration profile at the same spatial position. The plotted regulatory inputs were calculated at time class T6 and at position $x = 47.1\%$ EL, which is the center of the interval described in Fig. S14. The arrows mark the following three important stages at the response curve (from bottom to top): the input to *hb* expression from the rest of the gap genes is almost fully provided by activation from Gt and repression from Kni, this input is fully determined by repression from Kni alone (the rising repression from Kr compensates the decreasing activation from Gt at this point), and it is fully determined by repression from Kr and Kni.

that the main influence on *hb* expression is provided by proteins Kr and Kni for this Bcd concentration range. As the median Bcd case corresponds exactly to this Bcd range, this result is in agreement with earlier findings [2, 1, 3].

For the intermediate Bcd concentration values, the grey curve turns away from the red curve towards the orange one, and this is the key part of the regulatory curve for explaining the folds. Therefore, this part is associated with the two counteracting processes happening for rising Bcd concentration: the decrease of Gt influence and increase of Kr influence. We can see, in particular, how at the point marked by the intermediate arrow in the figure the activation from Gt is completely compensated by the repression from Kr, so that the grey line intersect the magenta one. The regulatory influence on *hb* from the three gap genes at this point is solely determined by repression from Kni.

This analysis is thus suggesting that Kni plays a passive role and is not responsible for the existence of folds. This conclusion was checked by the following computational experiment. We obtained solutions in the full model for all Bcd profiles but with an additional constraint that

$v_i^{\mathrm{Kni}}(t)$ was fixed equal to the Kni component of the solution in the median Bcd case. The *hb* response curve resulted from this experiment (Fig. S16A) is almost the same as the original one (Fig. 6B in the main text). The regulatory analysis in this case reveals the same mechanism of folding (Fig. S16B). The response curve in terms of regulatory inputs stays monotonously increasing with Bcd concentration as long as the main regulator is either Gt or Kr (with Kni excluded from the analysis) and turns to the decreasing form for Bcd concentration values associated with gradual shift from exclusive influence of Gt to exclusive influence of Kr.

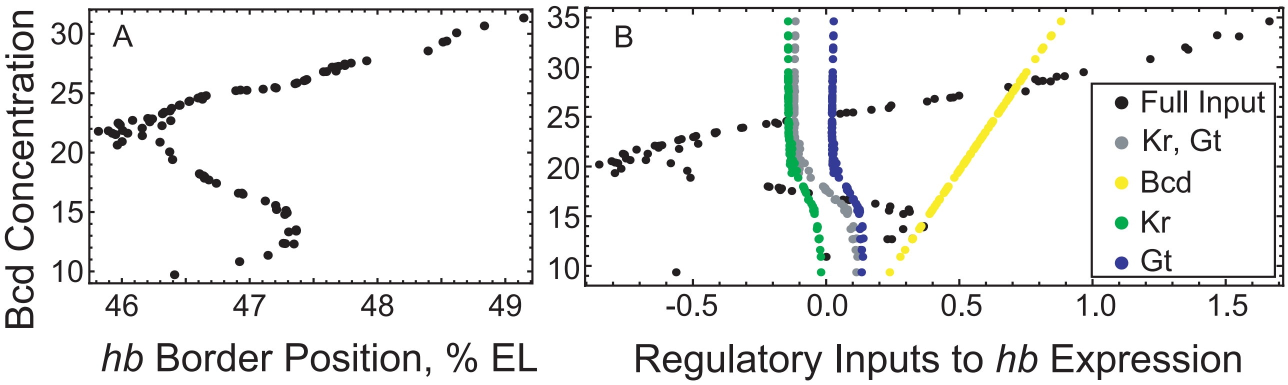

Figure S16. (A): The response curve (analog of Fig. 6B from the main text) resulted from solutions of the full model equations for all Bcd profiles but with the Kni component $v_i^{\mathrm{Kni}}(t)$ in these solutions artificially fixed equal to the Kni component of solution for the median Bcd profile. (B): The regulatory analysis of this response curve (analog of Fig. S15). As Kni is fixed and does not vary with varying Bcd, it is excluded from this analysis.

This shift can be explained by the fact that rising repression from Kr for increasing Bcd concentration is not compensated by any other factors. The anterior border of the central *Kr* expression domain (the *Kr* expression border nearest to the *hb* border) shifts always anteriorly for increasing Bcd concentration (Fig. S17B), until it fully vanishes for large enough Bcd concentrations with *Kr* highly expressed in the whole anterior region. On the other hand, the posterior border of the anterior *gt* expression domain (the *gt* expression border nearest to the *hb* border) exhibits the response curve which is asynchronous to the *hb* response curve (Fig. S17A), until it fully vanishes for large enough Bcd concentrations with no *gt* expression in the whole anterior region. This instability of the *Kr* and *gt* borders are due to missing regulators in the model acting at the anterior end [3].

References

[1] J. Jaeger, M. Blagov, D. Kosman, K. N. Kozlov, Manu, E. Myasnikova, S. Surkova, C. E. Vanario-Alonso, M. Samsonova, D. H. Sharp, and J. Reinitz. Dynamical analysis of regu-

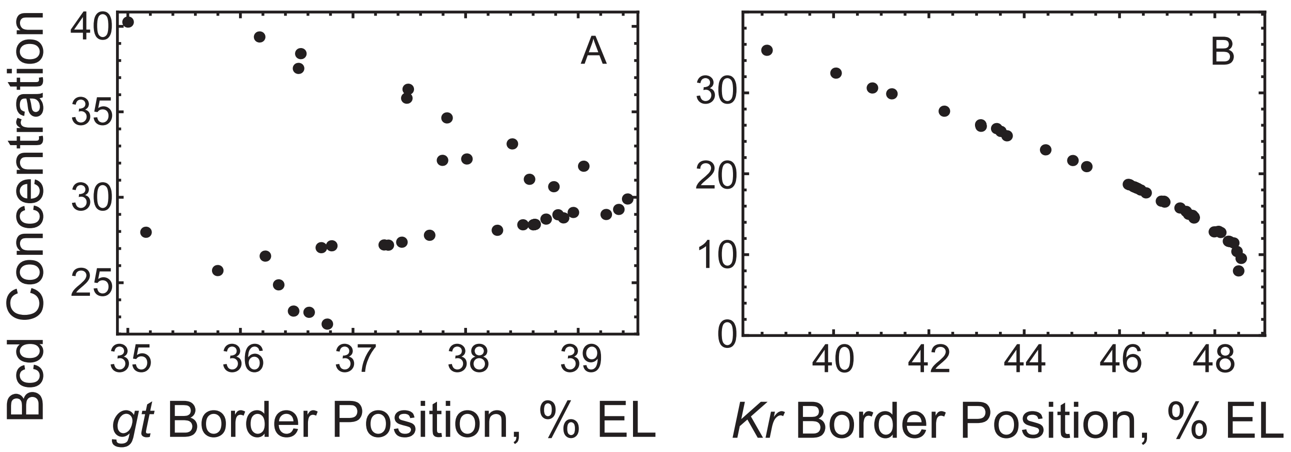

Figure S17. The response curves (analogs of Fig. 6B from the main text) for (A) the posterior border of anterior *gt* expression domain and (B) the anterior border of central *Kr* expression domain. Only 38 solutions (for 38 Bcd profiles) having these expression borders fully formed were analyzed.

latory interactions in the gap gene system of *Drosophila melanogaster*. *Genetics*, 167:1721–1737, 2004.

[2] J. Jaeger, S. Surkova, M. Blagov, H. Janssens, D. Kosman, K. N. Kozlov, Manu, E. Myasnikova, C. E. Vanario-Alonso, M. Samsonova, D. H. Sharp, and J. Reinitz. Dynamic control of positional information in the early *Drosophila* embryo. *Nature*, 430:368–371, 2004.

[3] Manu, S. Surkova, A. V. Spirov, V. Gursky, H. Janssens, A. Kim, O. Radulescu, C. E. Vanario-Alonso, D. H. Sharp, M. Samsonova, and J. Reinitz. Canalization of gene expression in the *Drosophila* blastoderm by gap gene cross regulation. *PLoS Biology*, 7:e1000049, 2009. doi:10.371/journal.pbio.1000049.

[4] J. Reinitz and D. H. Sharp. Mechanism of *eve* stripe formation. *Mechanisms of Development*, 49:133–158, 1995.
